# ATIR101 administered after T-cell-depleted haploidentical HSCT reduces NRM and improves overall survival in acute leukemia

**DOI:** 10.1038/s41375-020-0733-0

**Published:** 2020-02-11

**Authors:** Denis Claude Roy, Irwin Walker, Johan Maertens, Philippe Lewalle, Eduardo Olavarria, Dominik Selleslag, Sylvie Lachance, Marc Buyse, Kun Wang, Jeroen Rovers, Irene Santi, Halvard Bonig, Andrew Sandler, Jurjen Velthuis, Stephan Mielke

**Affiliations:** 1 Blood and Marrow Transplantation Program, Division of Hematology & Oncology, Hôpital Maisonneuve-Rosemont, Université de Montreal, Montreal, QC Canada; 2 Juravinski Hospital and Cancer Centre, McMaster University, Hamilton, ON Canada; 3 KU Leuven, Microbiology Immunology & Transplantation, Clinical Bacteriology and Mycology, and Department of Hematology, University Hospital Gasthuisberg, Leuven, Belgium; 4 Laboratory of Experimental Hematology, Jules Bordet Institut, Université Libre de Bruxelles, Bruxelles, Belgium; 5 Centre for Haematology, Imperial College London at Hammersmith Hospital, London, UK; 6 AZ Sint Jan Brugge-Oostende, Brugge, Belgium; 7 International Drug Development Institute, Louvain-la-Neuve, Belgium; 8 DC Prime, Leiden, Netherlands; 9 Kiadis Pharma, Amsterdam, Netherlands; 10 Institute for Transfusion Medicine and Immunohematology, Goethe University and German Red Cross Blood Service Baden-Württemberg-Hesse, Frankfurt, Germany; 11 Department of Internal Medicine II, Center of Allogeneic Stem Cell Transplantation, University of Wuerzburg, Wuerzburg University Medical Center, Wuerzburg, Germany; 12 Department of Laboratory Medicine, CAST, Karolinska Institute and University Hospital, Stockholm, Sweden

**Keywords:** Bone marrow transplantation, T cells

## Abstract

Overcoming graft-versus-host disease (GvHD) without increasing relapse and severe infections is a major challenge after allogeneic hematopoietic stem-cell transplantation (HSCT). ATIR101 is a haploidentical, naïve cell-enriched T-cell product, depleted of recipient-alloreactive T cells to minimize the risk of GvHD and provide graft-versus-infection and -leukemia activity. Safety and efficacy of ATIR101 administered after T-cell-depleted haploidentical HSCT (TCD-haplo + ATIR101) without posttransplant immunosuppressors were evaluated in a Phase 2, multicenter study of 23 patients with acute leukemia and compared with an observational cohort undergoing TCD-haplo alone (*n* = 35), matched unrelated donor (MUD; *n* = 64), mismatched unrelated donor (MMUD; *n* = 37), and umbilical cord blood (UCB; *n* = 22) HSCT. The primary endpoint, 6-month non-relapse mortality (NRM), was 13% with TCD-haplo + ATIR101. One year post HSCT, TCD-haplo + ATIR101 resulted in lower NRM versus TCD-haplo alone (*P* = 0.008). GvHD-free, relapse-free survival (GRFS) was higher with TCD-haplo + ATIR101 versus MMUD and UCB (both *P* < 0.03; 1-year rates: 56.5%, 27.0%, and 22.7%, respectively) and was not statistically different from MUD (1 year: 40.6%). ATIR101 grafts with high third-party reactivity were associated with fewer clinically relevant viral infections. Results suggest that haploidentical, selective donor-cell depletion may eliminate requirements for posttransplant immunosuppressors without increasing GvHD risk, with similar GRFS to MUD. Following these results, a randomized Phase 3 trial versus posttransplant cyclophosphamide had been initiated.

## Introduction

Despite recent translational achievements, allogeneic hematopoietic stem-cell transplantation (HSCT) still represents the only established curative option for most high-risk hematologic malignancies [1]. Human leukocyte antigen (HLA)-matched donors are the first choice, to reduce the risk of graft-versus-host disease (GvHD). Nonetheless, the timely availability of a matched unrelated donor (MUD), high incidences of chronic GvHD (cGvHD), and high relapse rates remain well-known obstacles to the overall improvement of outcome after allogeneic HSCT [2, 3]. As most patients have multiple potential haploidentical family donors, recent advances in both T-cell-replete and T-cell-depleted haploidentical HSCT have overcome donor shortage and are now challenging the standard use of matched HSCT without compromising outcomes [4–14]. In vivo depletion of alloreactive T cells with posttransplant cyclophosphamide (PTCY) is the most frequently utilized approach to haploidentical HSCT, yet severe GvHD still occurs despite use of immunosuppression and relapse remains an ongoing concern [15–19].

Haploidentical HSCT became feasible in the early 1990s after the development of extensive in vivo and ex vivo T-cell depletion, allowing stable engraftment across major HLA disparity in the absence of severe GvHD [15]. Nonetheless, overall outcomes were poor—the nonselective depletion of donor lymphocytes resulted in high rates of relapse and infections [15, 20]. This historical approach led to the development of strategies allowing reintroduction of different sources of T cells to improve posttransplant immune reconstitution, including gamma–delta T cells using alpha–beta T-cell-CD19 graft depletion, memory cells using CD45RA depletion, or infusion of unselected T cells under the control of suicide genes such as inducible caspase-9 or thymidine kinase [21–27]. Although these efforts are contributing to decreased transplant complications, there is no standard approach for haploidentical HSCT and there is still a need for improvement in control of infections, GvHD, and relapse.

Here, ATIR101 was used as a donor-derived, T-lymphocyte-enriched preparation selectively depleted of recipient-alloreactive T cells to minimize GvHD risk and maintain anti-infective and anti-leukemic activity [28–33]. ATIR101 is biologically depleted of host-reactive donor T cells through the ex vivo use of TH9402 and photodepletion [29]. The safety of ATIR101 was demonstrated in a Phase 1 study of T-cell-depleted haploidentical HSCT followed by ATIR101 (TCD-haplo + ATIR101) in high-risk patients. Grade 3/4 acute GvHD (aGvHD) was not observed at doses up to 5.0 × 10^6^ CD3^+^ cells/kg. At lower doses of 0.3–2.0 × 10^6^ cells/kg, there were no serious infections at 1 year and a transplant-related mortality of 0% with 67% stable survival after 8 years [34]. Consequently, safety, feasibility, and efficacy of ATIR101 at a dose of 2.0 × 10^6^ CD3^+^ cells/kg after TCD-haplo were evaluated in this single-arm, pivotal, and multicenter Phase 2 study (CR-AIR-007).

Based on the suggestion of regulators, an observational registry study (CR-AIR-006) served as a control and provided outcomes from MUD, mismatched unrelated donor (MMUD), double umbilical cord blood (UCB), and TCD-haplo HSCT without ATIR101. In this study, addition of ATIR101 to TCD-haplo significantly improved outcomes compared with TCD-haplo alone. Interestingly, a low aGvHD and cGvHD frequency in the absence of posttransplant immunosuppressors and a low relapse rate resulted in 1-year GvHD-free, relapse-free survival (GRFS) not being significantly different from MUD HSCT. The evaluation of ATIR101 cells also demonstrated naïve T-cell enrichment. Moreover, an increased level of anti-third-party reactivity in donor T cells (possibly indicating “T-cell fitness”) was associated with lower rates of severe viral infections.

## Methods

### Study design

CR-AIR-007 was designed as a pivotal Phase 2, single-arm, exploratory, open-label, and multicenter study to evaluate the safety, feasibility, and efficacy of ATIR101 in patients receiving TCD-haplo in centers in Europe and North America (“ATIR101 study”; NCT01794299). The primary endpoint was non-relapse mortality (NRM) at 6 months post HSCT, defined as death due to causes other than disease relapse or progression, or other causes unrelated to transplantation. An interim analysis was planned for when ten patients had been treated with ATIR101 and followed up for 6 months after HSCT. Patient accrual was to be stopped if the number of NRM cases within 6 months post HSCT exceeded 4 among the first 10 or 6 among the first 23 patients receiving ATIR101. Secondary endpoints included: immune reconstitution; NRM; relapse-related mortality (RRM); overall survival (OS); progression-free survival (PFS); incidence and severity of aGvHD, cGvHD, and infections until the end of follow-up at 2 years.

CR-AIR-006 was designed based on suggestions of regulators as an observational registry study (“control study”; NCT02188290) to provide valid, relevant, external control groups for comparison with ATIR101-treated patients without randomization. Therefore, a restricted cohort design was applied to adapt the design of an observational study to the principles of randomized controlled trials (by identifying a baseline to determine patient eligibility, using inclusion/exclusion criteria similar to those in ATIR101 studies; and by adjusting for relevant prognostic factors and using statistical methods similar to those used in randomized controlled trials) [35, 36]. Data were collected prospectively by centers as part of an international registry on HSCT (e.g., EBMT or CIBMTR). The aim was to provide a complete, anonymous inventory of all eligible patients meeting inclusion/exclusion criteria at each center in a web-based database system (ProMISe). NRM (defined as deaths that could not be attributed to disease relapse or progression as in the ATIR101 study), RRM, OS, PFS, and the incidence and severity of GvHD were endpoints of this control study with a planned follow-up of 1 year after HSCT. GRFS was a post hoc composite exploratory endpoint in both studies (see Supplementary information) [37].

Patients aged 18–65 years with acute myeloid leukemia or acute lymphoblastic leukemia in remission or with myelodysplastic syndrome without a prior stem-cell transplantation were eligible for both studies. See Supplementary information for more detailed information.

### ATIR101 study (CR-AIR-007)

Patients with a haploidentical donor with two or three mismatches at the HLA-A, -B, and/or -DR loci of the unshared haplotype, without a timely available suitable matched donor were candidates for the ATIR101 study.

**Fig. 1:**
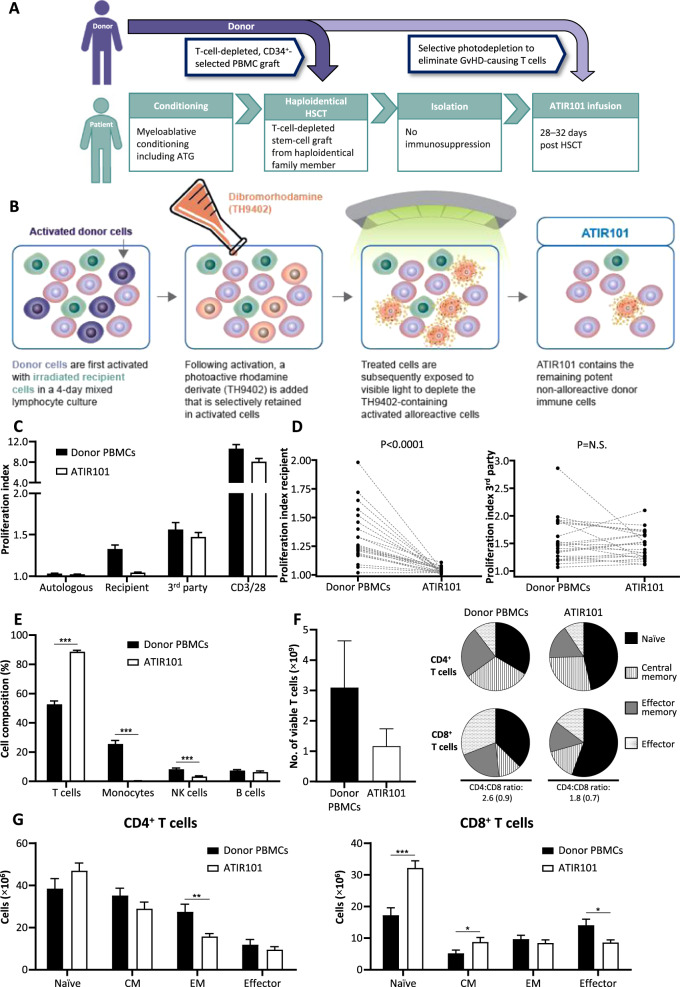
ATIR101 characterization and infusion. **a** Schematic overview of the treatment of patients with ATIR101. Donor and patient PBMCs, as well as donor plasma, are obtained for production of ATIR101. The same donor is thereafter treated with G-CSF to mobilize stem cells and collect the graft. Patients then undergo myeloablative conditioning including ATG followed by HSCT with the CD34^+^-selected graft. ATIR101 infusion was planned for 28–32 days post HSCT. Patients do not receive posttransplant immunosuppression. **b** Schematic overview of the manufacturing process of ATIR101. Donor and patient lymphocytes are collected via apheresis and isolated over density gradient. Patient lymphocytes only are then irradiated and cultured together with donor lymphocytes for 4 days. During this period, donor anti-host alloreactive T cells are activated due to the presence of “foreign” HLAs on the patient’s cells. Next, 4,5-dibromorhodamine methyl ester (TH9402) is added to the culture. TH9402 is selectively retained in activated cells due to low P-glycoprotein activity responsible for its extrusion into the outside environment. After exposure to light, the dye becomes activated and a source of the reactive oxygen species, which, at high concentrations, leads to cell apoptosis. The remaining cells are infused into the patient (ATIR101). Adapted from figure available at: https://www.kiadis.com/products-and-technology/ (accessed May 2019). **c** CFSE-based proliferation assay to calculate the T-cell proliferation index in response to various stimuli. Untreated donor PBMCs (black bars) and ATIR101 cells (white bars) were stimulated with (1) irradiated autologous donor PBMCs to determine baseline proliferation by adding cells that may provide a “feeder effect”; (2) irradiated recipient PBMCs to determine recipient-specific activity; (3) irradiated third-party cells to determine activity against unrelated HLA; (4) anti-CD3/CD28 beads to determine overall proliferative capacity of T cells. Data are presented as mean ± standard error of the mean. **d** T-cell proliferation index of individual donor PBMCs and corresponding ATIR101 cells upon exposure to irradiated recipient T cells (left) and third-party (right) cells. **e** Mean proportion (± standard error of the mean) of T cells, monocytes, NK cells, and B cells in ATIR101 (white bars) compared with donor PBMCs (black bars) by flow cytometry. **f** Mean absolute number (± standard error of the mean) of viable T cells in the starting volume of donor PBMCs by flow cytometry and a theoretical equivalent volume of ATIR101. Pie charts: mean proportion (%) of CD4^+^ and CD8^+^ naïve (black), central memory (striped), effector memory (gray), and effector (dots) T cells and the average CD4:CD8 ratio (standard deviation) of donor PBMCs and ATIR101 by flow cytometry. **g** Mean absolute number (± standard error of the mean) of CD4^+^ and CD8^+^ naïve, CM, EM, and effector T cells. The figure shows absolute number of each cell type infused within a 2 × 10^6^ CD3^+^ cells/kg dose of ATIR101 (white bars) and within a representative equivalent sample of 2 × 10^6^ CD3^+^ cells/kg of donor PBMCs (black bars). *P < 0.05, **P < 0.01, ***P < 0.001 for ATIR101 compared with donor PBMCs. ATG anti-thymocyte globulin, CFSE carboxyfluorescein succinimidyl ester, CM central memory, EM effector memory, G-CSF granulocyte colony-stimulating factor, GvHD graft-versus-host disease, HLA human leukocyte antigen, HSCT hematopoietic stem-cell transplantation, NK natural killer, N.S. not significant, PBMC peripheral blood mononuclear cell.

Before donor granulocyte colony-stimulating factor treatment and graft collection, donor and patient peripheral blood mononuclear cells (PBMCs) were obtained by apheresis for ATIR101 production, which is described in Fig. 1b and the Supplementary information. Collection and preparation of the donor peripheral blood stem-cell graft were performed according to center procedures (Supplementary information). All patients received thiotepa, fludarabine, anti-thymocyte globulin, and total body irradiation or melphalan myeloablative conditioning (Fig. 1a; Supplementary information). No patient received post-HSCT immunosuppressant GvHD prophylaxis. Engraftment was defined as neutrophils ≥0.5 × 10^9^/L for two consecutive days and platelets ≥20 × 10^9^/L for three consecutive days, without transfusion.

### Characterization of ATIR101 and donor PBMCs in the ATIR101 study

The characterization of ATIR101 and donor PBMCs included phenotyping for the proportion of T cells (CD3^+^), monocytes (CD14^+^), B cells (CD19^+^), and natural killer (NK) cells (CD3^−^/CD16^+^CD56^+^) and determination of memory T-cell subsets (CD45RO and CD62L) by multiparameter flow cytometry (Supplementary information). A carboxyfluorescein succinimidyl ester-dilution-based proliferation assay was used to determine the specificity of depletion and the immunologic potency of remaining ATIR101 cells and donor PBMCs. Dextramer stainings were performed to determine the number of CD8^+^ viral (Epstein–Barr virus [EBV]/cytomegalovirus [CMV]) dextramer-positive cells. ELISA assays were used to measure IFNy production from re-stimulated total donor PBMCs and ATIR101 (either with CMV, EBV, or pathomix). See Supplementary information for more detailed information.

### Control study (CR-AIR-006)

Planned control groups included patients undergoing: TCD-haplo (1 January 2006–30 June 2013); MUD/1-locus MMUD (1 January 2010–31 December 2012); double UCB (1 January 2010–31 December 2012). Data were collected at European/North American centers that enrolled patients in ATIR101 clinical studies. Data were collected from all eligible patients in the TCD-haplo group (and all patients in other groups if they had ≤75 eligible patients), otherwise random selection was performed in Structured Query Language directly on the study database until the following was met: sample size of 60–70; each center contributed ≥1 patient for each diagnosis (except for Montréal, where no patients with myelodysplastic syndrome diagnosis were included); overall ratio of diagnoses corresponded to the ratio in the TCD-haplo group. Information was collected in a study-specific case report form for the selected patients. Patient and donor information collected is listed in the Supplementary information. Definition of engraftment was similar to the ATIR101 study (absolute neutrophil count >0.5 × 10^9^/L for two consecutive days; platelets >20 × 10^9^/L for three consecutive days, without transfusion).

### Statistics

The Wilcoxon signed-rank test was used to compare characteristics of ATIR101 batches with statistical significance level set at *P* ≤ 0.05 (two sided), since the aim was to test for differences between two related batches (so variance is the same) but the assumptions of a paired sample *t* test are violated (difference between repeated measurements are not normally distributed, or if outliers exist). Rates of time-to-event endpoints and cumulative summary of GvHD and relapse at 6 and 12 months were calculated. The Kaplan–Meier (KM) method stratified by the type of hematologic malignancy was also used to estimate the time-to-event endpoints (OS, PFS, GRFS) and groups compared using log-rank test (two sided) with Bonferroni correction for multiple comparisons. Hazard ratios (HRs) and their corresponding 95% confidence intervals (CIs) were also calculated. The KM method was used to evaluate time-to-Grade ≥3 viral infections with groups compared using the log-rank test. NRM, RRM, and GvHD were assessed using the cumulative incidence function for competing risks, which included RRM, NRM, and death without GvHD, respectively; differences between groups were determined using Gray’s test [38]. Statistical analyses of ATIR101 characteristics were performed with GraphPad Prism v7. SAS software (v9.3) was used for the remaining analyses.

## Results

### Patient characteristics

A total of 158 patients from 9 European and North American centers were included in the control study. They received transplants between February 2006 and May 2013 from haploidentical donors with T-cell depletion (*n* = 35), MUD (*n* = 64), MMUD (*n* = 37), or UCB (*n* = 22; Supplementary Fig. 1). Between March 2013 and September 2017, a total of 26 patients underwent HSCT in CR-AIR-007 (intention-to-treat [ITT] population) from 7 of the same centers included in the control study. Twenty-three patients received ATIR101 (TCD-haplo + ATIR101). Three patients did not receive ATIR101 due to early death of the patient, production failure, and primary graft failure. Of patients receiving TCD-haplo + ATIR101, 70% had acute myeloid leukemia and 30% had acute lymphoblastic leukemia, with 57% categorized as high disease risk index [39]. Although eligible for both studies, no patients with MDS were enrolled into the ATIR101 study. However, the distribution of disease categories in the TCD-haplo + ATIR101 study was not statistically significantly different from that in the control study (Kruskall–Wallis *P* = 0.9713). Patient characteristics are presented in Tables 1 and 2.

**Table 1:** Patient characteristics in the ATIR101 study.

	ITT population *N* = 26	TCD-haplo + ATIR101 *N* = 23
Median age, years (range)	43 (20–64)	41 (21–64)
Sex, male, *n* (%)	14 (53.8)	12 (52.2)
AML *n* (%)	19 (73.1)	16 (69.6)
CR1	14 (73.7)	11 (68.8)
CR2	5 (26.3)	5 (31.3)
ALL, *n* (%)	7 (26.9)	7 (30.4)
CR1	4 (57.1)	4 (57.1)
CR2	3 (42.9)	3 (42.9)
Disease risk index^a^		
Intermediate	13 (50.0)	10 (43.5)
High	13 (50.0)	13 (56.5)
Conditioning regimen, *n* (%)		
Myeloablative	26 (100)	23 (100)
TBI	12 (46.2)	11 (47.8)
Anti-thymocyte globulin	26 (100)	23 (100)
Donor median age, years (range)	33 (20–60)	34 (20–60)
Donor sex, male, *n* (%)	12 (46.2)	10 (43.5)
Donor type, *n* (%)		
Parent	5 (19.2)	4 (17.4)
Sibling	9 (34.6)	9 (39.1)
Child	11 (42.3)	9 (39.1)
Other family member	1 (3.8)	1 (4.3)
HLA-A, -B, -DR, *n* (%)		
3/6	19 (73.1)	16 (69.6)
4/6	6 (23.1)	6 (26.1)
5/6	1 (3.8)^b^	1 (4.3)^b^
CMV status, donor/patient, *n* (%)		
+/+	11 (42.3)	8 (34.8)
+/−	2 (7.7)	2 (8.7)
–/+	3 (11.5)	3 (13.0)
–/–	10 (38.5)	10 (43.5)
EBV status, donor/patient, *n* (%)		
+/+	23 (88.5)	20 (87.0)
+/–	1 (3.8)	1 (4.3)
–/+	0	0
–/–	2 (7.7)	2 (8.7)
CD34^+^-selected graft		
CD34^+^ × 10^6^/kg, median (range)	11.0 (3.2–24.4)	11.0 (4.7–24.4)
CD3^+^ × 10^4^/kg, median (range)^c^	0.31 (0.01–1.8)	0.29 (0.01–1.8)
Engraftment		
Platelets, median days (range)	11 (9–35)	11 (9–35)
Neutrophils, median days (range)	12 (8–34)	12 (8–34)

*ALL* acute lymphoblastic leukemia, *AML* acute myeloid leukemia, *CMV* cytomegalovirus, *CR* complete remission, *EBV* Epstein–Barr virus, *HLA* human leukocyte antigen, *ITT* intention-to-treat, *TBI* total body irradiation, *TCD-haplo* T-cell-depleted haploidentical hematopoietic stem-cell transplantation.

^a^Disease risk index was calculated on available data of the disease (AML/ALL, cytogenetics, molecular abnormalities) and disease status (first, second, or later CR).

^b^This patient had a 7/10 HLA match on the HLA-A, -B, -C, -DQ, and -DR loci.

^c^Based on known doses.

**Table 2:** Patient characteristics in the TCD-haplo + ATIR101 population and the control cohorts.

Characteristics	TCD-haplo + ATIR101 *N* = 23	Control study			
		TCD-haplo *N* = 35	MUD *N* = 64	MMUD *N* = 37	UCB *N* = 22
Median age, years (range)	41 (21–64)	43 (19–62)	47.5 (20–63)	54 (28–65)	38.5 (18–64)
Sex, male, *n* (%)	12 (52.2)	20 (57.1)	34 (53.1)	14 (37.8)	12 (54.5)
AML, *n* (%)	16 (69.6)	25 (71.4)	43 (67.2)	25 (67.6)	14 (63.6)
CR1	11 (68.8)	18 (72.0)	32 (74.4)	15 (60.0)	9 (64.3)
CR2	5 (31.3)	4 (16.0)	10 (23.3)	7 (28.0)	3 (21.4)
>CR2	0	3 (12.0)^a^	0	2 (8.0)	2 (14.3)
Unknown	0	0	1 (2.3)	1 (4.0)	0
ALL, *n* (%)	7 (30.4)	4 (11.4)	9 (14.1)	7 (18.9)	5 (22.7)
CR1	4 (57.1)	1 (25.0)	5 (55.6)	5 (71.4)	4 (80.0)
CR2	3 (42.9)	3 (75.0)	4 (44.4)	1 (14.3)	1 (20.0)
>CR2	0	0	0	0	0
Unknown	0	0	0	1 (14.3)	0
MDS, *n* (%)	0	6 (17.1)	12 (18.8)	5 (13.5)	3 (13.6)
Preparative regimen, *n* (%)					
Myeloablative	23 (100)	33 (94.3)	34 (53.1)	21 (56.8)	13 (59.1)
TBI	11 (47.8)	23 (65.7)	29 (45.3)	15 (40.5)	22 (100)
Anti-thymocyte globulin	23 (100)	34 (97.1)	44 (68.8)	30 (81.0)	1 (4.5)
Donor type, *n* (%)					
Parent	4 (17.4)	8 (22.9)	0	0	0
Sibling	9 (39.1)	15 (42.9)	0	0	0
Child	9 (39.1)	10 (28.6)	0	0	0
Other family member	1 (4.3)	2 (5.7)	0	0	0
Unrelated	0 (0)	0	64 (100)	37 (100)	22 (100)
Graft cell dose infused					
CD34^+^ × 10^6^/kg, median (range)	11.0 (4.7–24.4)	7.4 (4.6–10.1)	7 (1.5–920)	6.6 (0.6–390)	0.14 (0.0–13.7)
CD3^+^ × 10^4^/kg, median (range)^b^	0.29 (0.01–1.8)	2.4 (0.6–5.0)^c^	9030 (1.5–50000)	4600 (0.7–39900)	911 (834–1200)
Engraftment (days)					
Platelets, median (range)	11 (9–35)	20 (5–67)	18 (8–173)	16 (9–44)	39 (31–130)
Neutrophils, median (range)	12 (8–34)	17 (10–38)	17 (9–37)	17 (9–25)	20 (2–45)

*ALL* acute lymphoblastic leukemia, *AML* acute myeloid leukemia, *CR* complete remission, *MDS* myelodysplastic syndrome, *MMUD* mismatched unrelated donor, *MUD* matched unrelated donor, *PR* partial remission, *TBI* total body irradiation, *TCD-haplo* T-cell-depleted haploidentical hematopoietic stem-cell transplantation, *UCB* umbilical cord blood.

^a^One of these patients was in PR (having two prior remissions).

^b^Based on known doses.

^c^Data from 26 patients.

### HSCT and ATIR101

Patients treated with TCD-haplo + ATIR101 received a CD34^+^-selected allograft containing a median of 11.0 × 10^6^ CD34^+^ cells/kg (range: 4.7–24.4) and a residual amount of T cells (median 0.29 × 10^4^ CD3^+^ cells/kg; range 0.01–1.8). Neutrophil and platelet engraftment occurred at a median of 12 (range 8–34) and 11 (range 9–35) days after HSCT, respectively (Table 1). All patients received an ATIR101 dose of 2.0 × 10^6^ viable T cells/kg at a median of 28 days (range 28–73) post HSCT (Supplementary information). Figure 1a illustrates the treatment process and Fig. 1b the principle of ATIR101 production. After this selective allodepletion process, responses against third party and polyclonal stimulators were maintained, while patient-specific alloreactivity was significantly reduced in all products (Fig. 1c, d). T cells were relatively enriched in ATIR101 (89% ± 4.7) compared with donor PBMCs (53% ± 10.3; Fig. 1e; *P* < 0.001). The proportion of monocytes and NK cells was significantly lower in ATIR101 than in donor PBMCs (both *P* < 0.001), while some B cells were retained. Evaluation of T-cell subsets suggested that the proportion of CD4^+^ and CD8^+^ naïve cells was higher in ATIR101 than in donor PBMCs (Fig. 1f), and subsequent analysis showed a statistically significant absolute increase in the CD8^+^ compartment of ATIR101 (Fig. 1g; *P* < 0.001). Interestingly, there was an absolute increase in central memory T cells and a reduction of the CD8^+^ effector-cell subset versus donor PBMCs (both *P* < 0.05; Fig. 1g). Overall, the CD4^+^:CD8^+^ ratio remained comparable between ATIR101 and donor PBMCs (Fig. 1f).

### Immune reconstitution after TCD-haplo+ATIR101

**Fig. 2:**
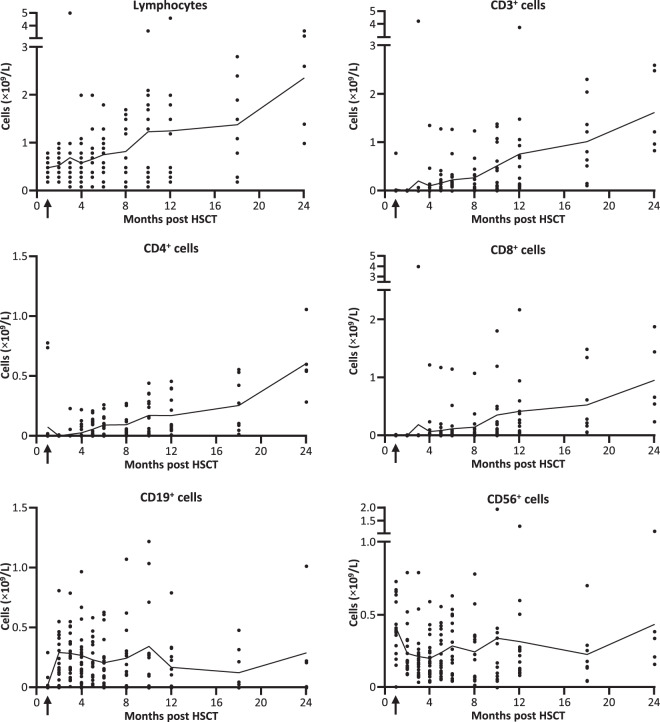
Immune reconstitution in the TCD-haplo + ATIR101 population (*N* = 23). Absolute number of lymphocytes, T cells (CD3^+^, CD4^+^, and CD8^+^), B cells (CD19^+^), and NK cells (CD56^+^) as cells ×10^9^/L in individual patient samples to 24 months post HSCT. Line shows mean values. Arrow indicates time of ATIR101 infusion. HSCT hematopoietic stem-cell transplantation.

Lymphocyte recovery was highly variable, with median lymphocyte and CD3^+^ T-cell levels continuously rising from early after transplantation until 24 months (Fig. 2). From 6 months onwards, the mean CD3^+^ count was above the level indicative of minimal reconstitution of cellular immunity (0.2 × 10^9^/L). NK and B cells recovered early post HSCT. Further subpopulation analyses are provided in the Supplementary information.

### NRM, RRM, PFS, and OS

**Fig. 3:**
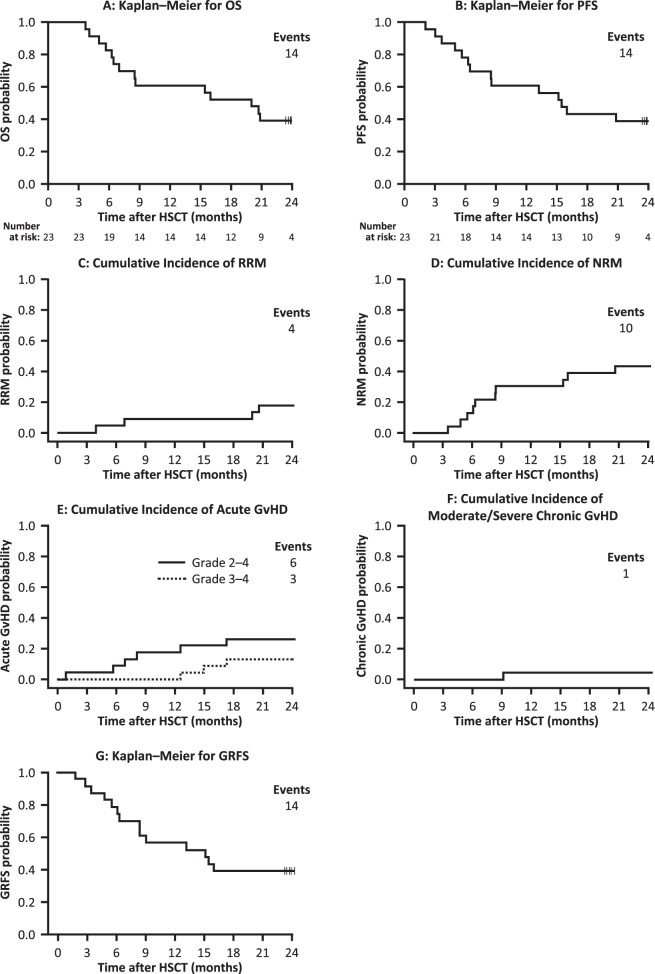
Outcomes following TCD-haplo + ATIR101 (*N* = 23). Kaplan–Meier plots are shown for OS (**a**), PFS (**b**), and GRFS (**g**). Cumulative incidence plots, taking into account competing risks, for RRM (**c**), NRM (**d**), acute GvHD Grade 2–4 and Grade 3–4 (**e**), and moderate/severe chronic GvHD (**f**). GRFS GvHD-free, relapse-free survival, GvHD graft-versus-host disease, HSCT hematopoietic stem-cell transplantation, NRM non-relapse mortality, OS overall survival, PFS progression-free survival, RRM relapse-related mortality.

The primary endpoint of 6-month NRM occurred in three patients receiving TCD-haplo + ATIR101 (rate 13.0%; Table 3). At 2 years, the OS rate was 39.1%, with ten deaths due to NRM (43.5%) and four due to relapse (17.4%; Fig. 3). Similar outcomes were observed in the ITT population (Supplementary Table 1; Supplementary Fig. 2). Of note, four patients received an unmanipulated donor leukocyte infusion (DLI) 4–127 days before NRM, which may have contributed to the rate of NRM at 2 years, and the protocol was later amended to restrict unmanipulated DLI usage for impending relapse or graft failure only. Unmanipulated DLI was used to treat infection in three patients or for the management of low lymphocyte count in a patient without any complication (Supplementary Table 2). Two further patients received unmanipulated DLI for the treatment of relapse. Two of the four patients with RRM relapsed beyond 12 months, resulting in a 2-year PFS rate of 39.1% (Fig. 3).

**Table 3:** Survival, NRM, infections, GvHD, relapse, GRFS, and PFS with TCD-haplo + ATIR101 and the control cohorts (point estimates).

	TCD-haplo + ATIR101 *N* = 23	Control study			
		TCD-haplo (*N* = 35)	MUD (*N* = 64)	MMUD^a^ (*N* = 37)	UCB (*N* = 22)
Patients with NRM event, *n* (%)					
6 months	3 (13.0)	13 (37.1)	4 (6.3)	8 (21.6)	7 (31.8)
12 months	7 (30.4)	23 (65.7)	6 (9.4)	9 (24.3)	8 (36.4)
Grade 2–4 acute GvHD (cumulative), *n* (%)^b^					
6 months	2 (8.7)	7 (20.0)	15 (23.4)	10 (27.0)	11 (50.0)
12 months	4 (17.4)	7 (20.0)	16 (25.0)	10 (27.0)	11 (50.0)
Grade 3–4 acute GvHD (cumulative), *n* (%)^b^					
6 months	0 (0.0)	2 (5.7)	6 (9.4)	6 (16.2)	6 (27.3)
12 months	0 (0.0)	2 (5.7)	7 (10.9)	6 (16.2)	6 (27.3)
Chronic GvHD (cumulative), *n* (%)^b^					
6 months	0 (0.0)	3 (8.6)	12 (18.8)	4 (10.8)	5 (13.5)
12 months	1 (4.3)	3 (8.6)	24 (37.5)	10 (27.0)	7 (31.8)
Relapse (cumulative), *n* (%)					
6 months	2 (8.7)	4 (11.4)	5 (7.8)	5 (13.5)	2 (9.1)
12 months	2 (8.7)	7 (20.0)	9 (14.1)	6 (16.2)	2 (9.1)
Patients with RRM event,^a^ *n* (%)					
6 months	1 (4.3)	0 (0.0)	2 (3.1)	2 (5.4)	1 (4.5)
12 months	2 (8.7)	5 (14.3)	3 (4.7)	4 (10.8)	2 (9.1)
PFS, *n* (%)					
6 months	18 (78.3)	20 (57.1)	55 (85.9)	24 (64.9)	13 (59.1)
12 months	14 (60.9)	7 (20.0)	49 (76.6)	22 (59.5)	12 (54.5)
GRFS, *n* (%)					
6 months	18 (78.3)	20 (57.1)	40 (62.5)	17 (45.9)	6 (27.3)
12 months	13 (56.5)	7 (20.0)	26 (40.6)	10 (27.0)	5 (22.7)
Overall survival, *n* (%)					
6 months	19 (82.6)	22 (62.9)	58 (90.6)	27 (73.0)	14 (63.6)
12 months	14 (60.9)	7 (20.0)	55 (85.9)	24 (64.9)	12 (54.5)

*GRFS* GvHD-free, relapse-free survival (acute GvHD Grade 3/4, chronic GvHD requiring systemic use of immunosuppressive medication, relapse, or death, whichever comes first in the first post-HSCT year), *GvHD* graft-versus-host disease, *MMUD* mismatched unrelated donor, *MUD* matched unrelated donor, *NRM* non-relapse mortality, *PFS* progression-free survival, *RRM* relapse-related mortality, *TCD-haplo* T-cell-depleted haploidentical hematopoietic stem-cell transplantation, *UCB* umbilical cord blood.

^a^As assessed by investigator.

^b^Each patient is represented with the maximum severity (2 and 3 patients in the MUD and MMUD groups, respectively, with acute GvHD of unknown severity are not captured here).

**Fig. 4:**
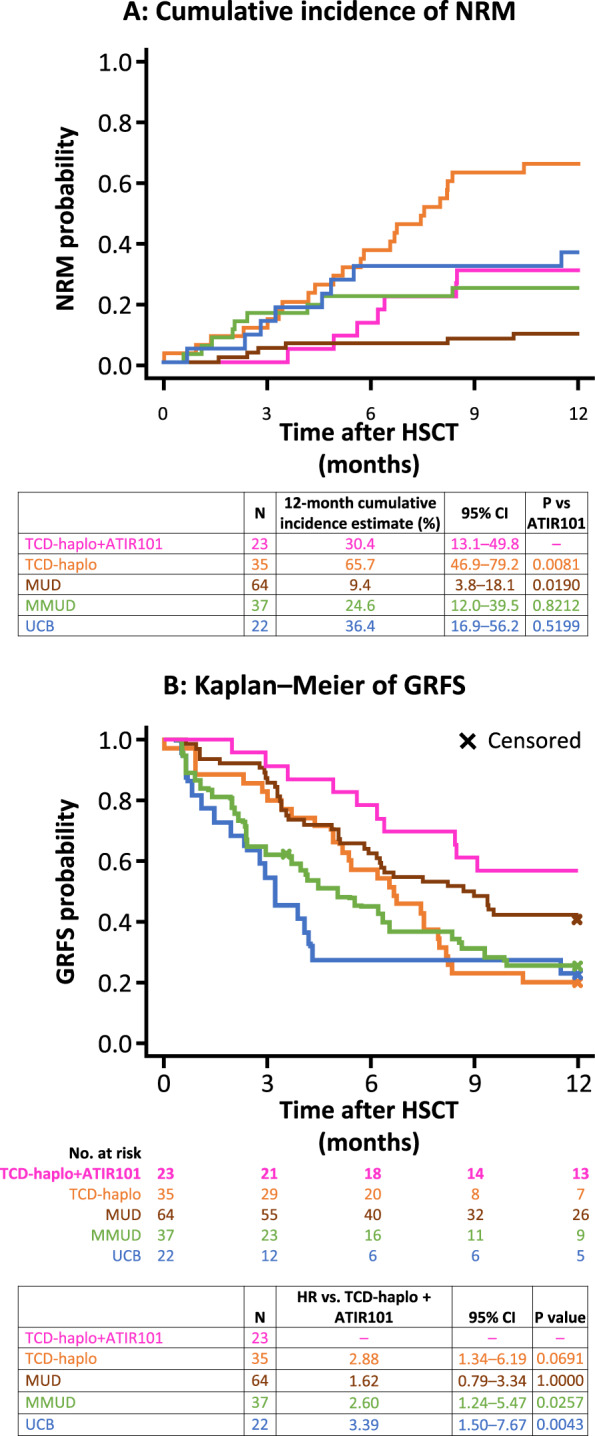
Cumulative incidence of NRM and Kaplan–Meier of GRFS for patients receiving TCD-haplo + ATIR101 (*N* = 23) and the control study TCD-haplo (*N* = 35), MUD (*N* = 64), MMUD (*N* = 37), and UCB (*N* = 22) populations. **a** Cumulative incidence plot of NRM taking into account competing risk and (**b**) Kaplan–Meier of GRFS over 1 year, for TCD-haplo + ATIR101 (pink) and patients from the control study who received TCD-haplo (orange), MUD (brown), MMUD (green), or double UCB (blue). **a** Groups were compared using Gray’s test. **b** HR and their corresponding 95% CI are presented, and groups are compared using the log-rank test with Bonferroni correction for multiple comparisons. CI confidence interval, GRFS graft-versus-host disease-free, relapse-free survival, HR hazard ratio, HSCT hematopoietic stem-cell transplantation, MMUD mismatched unrelated donor, MUD matched unrelated donor, NRM non-relapse mortality, TCD-haplo T-cell-depleted haploidentical HSCT, UCB umbilical cord blood.

Compared with TCD-haplo + ATIR101, the 6-month NRM rate was approximately threefold higher in the TCD-haplo alone group (13.0% and 37.1%, respectively; Table 3). Similarly, 6-month OS rate was lower with TCD-haplo (62.9%) than with TCD-haplo + ATIR101 (82.6%). The 12-month OS rate was 60.9% with TCD-haplo + ATIR101, including seven NRM (30.4%) and two RRM (8.7%). By comparison, the 12-month OS rate for TCD-haplo alone was only 20.0%, including 23 NRM (65.7%) and 5 RRM (14.3%), resulting in a significantly higher NRM versus TCD-haplo + ATIR101 (Fig. 4a; *P* = 0.008). There was also a more than threefold improvement in estimated OS with the addition of ATIR101 to TCD-haplo (HR: 3.10; 95% CI: 1.40–6.84; *P* = 0.0002; Supplementary Fig. 3A). In a subgroup analysis of the TCD-haplo alone group, limited to patients with AML and ALL (excluding MDS patients), similar differences were observed with 6- and 12-month NRM of 37.9% and 75.5%, and OS of 62.1% and 17.2%, respectively.

The primary cause of NRM was infection in both TCD-haplo populations; nevertheless, the 12-month cumulative incidence of infection-related NRM with TCD-haplo + ATIR101 was nearly half that of TCD-haplo alone (21.7% vs 40.0%, respectively). Grade ≥3 viral infections/reactivations occurred in 11 patients receiving TCD-haplo + ATIR101 (20 events) over 2 years (Supplementary Table 3). No Grade ≥3 viral infections/reactivations occurred during the period from HSCT to ATIR101 infusion (Supplementary Table 4). Between HSCT and ATIR101 infusion, viral, fungal, and bacterial infections had approximately the same incidence (35–39%). In later time intervals, viral infections prevailed over other infections. Among 11 CMV sero-positive patients, ten tested CMV positive by PCR post HSCT (including 2/3 with a negative donor); however, only one reported symptomatic CMV infection and none of the patients who were baseline CMV negative became CMV positive. Although 91% were at risk, only 39% experienced adverse events indicative of EBVs reactivation (eight positive patients at baseline and one negative patient with a positive donor). EBV resolved in 6/9 patients: five resolved after treatment and one spontaneously.

Seven patients receiving TCD-haplo alone relapsed by 12 months post HSCT (20% cumulative rate), which was more than double the 12-month rate with TCD-haplo + ATIR101 (8.7%; Table 3). In line with OS, the 12-month PFS rate was 60.9% with TCD-haplo + ATIR101 and 20.0% with TCD-haplo alone, with a more than threefold improvement in estimated PFS with the addition of ATIR101 (HR: 3.17; 95% CI: 1.43–7.02; *P* = 0.001; Supplementary Fig. 3B).

One-year cumulative incidence of NRM with TCD-haplo + ATIR101 (30.4%) was similar to MMUD (24.6%) and UCB (36.4%; Fig. 4a; both *P* > 0.5). The 1-year cumulative rate of relapse was 16.2% with MMUD and 9.1% with UCB (8.7% with TCD-haplo + ATIR101). There was also no statistically significant difference in cumulative incidence of RRM or estimated PFS and OS with TCD-haplo + ATIR101 versus MMUD and UCB (all *P* > 0.7; Supplementary Fig. 3). The 1-year cumulative incidence of NRM was significantly lower with MUD (9.4%) than with TCD-haplo + ATIR101 (30.4%; Fig. 4a; *P* = 0.019). Estimated OS and PFS were higher with MUD than TCD-haplo + ATIR101 (95% CI: 0.11–0.82, *P* = 0.003, Supplementary Fig. 3A; and 95% CI: 0.48–1.18, *P* = 0.043, Supplementary Fig. 3B, respectively). Although the 12-month cumulative relapse rate was 14.1% with MUD (Table 3), only 3/9 relapsed patients died in the first year due to relapse, explaining the lack of statistical difference in the cumulative incidence of RRM versus TCD-haplo + ATIR101 (4.7% and 8.7%, respectively; *P* = 0.482; Supplementary Fig. 3C). Comparisons were similar with the ATIR101 study ITT population (Supplementary Fig. 4).

### Graft-versus-host disease (GvHD)

Within the first year after HSCT, GvHD occurred in 15 (42.9% cumulative rate) of the control patients who received TCD-haplo alone, including 5 with Grade 2 aGvHD (14.3%), 2 with Grade 3/4 aGvHD (5.7%), and 3 with cGvHD (8.6%; Table 3). In the TCD-haplo + ATIR101 population, the cumulative 1-year rate of GvHD was 30.4% (seven patients), including three patients with Grade 1, four patients with Grade 2 (17.4%), and no Grade 3/4 aGvHD (Table 3). One patient with Grade 1 aGvHD later developed moderate cGvHD (4.3%) in the first year. Thus, addition of ATIR101, even in the absence of posttransplant immunosuppression, did not increase the 1-year cumulative incidence of Grade 2–4 aGvHD, Grade 3/4 aGvHD, or cGvHD (Supplementary Fig. 3D–F) over TCD-haplo alone. In contrast, the rate of Grade 3/4 aGvHD was 10.9%, 16.2%, and 27.3% with MUD, MMUD, and UCB, respectively (Table 3; *P* < 0.05 for ATIR101 versus UCB and MMUD; Supplementary Fig. 3F); notably, these occurred despite GvHD prophylaxis, whereas ATIR101 is given without any posttransplant immunosuppression. In addition, compared with TCD-haplo + ATIR101, cGvHD was significantly higher with MUD (37.5%), MMUD (27.0%), and UCB (31.8%; all *P* < 0.05; Supplementary Fig. 3D).

At 2 years, GvHD was reported in nine patients (39.1% cumulative rate; 19 events) receiving TCD-haplo + ATIR101: eight with aGvHD only (maximum Grade 2: 13.0%; Grade 3: 8.7%; Grade 4: 4.3%) and one with Grade 1 aGvHD and moderate/severe cGvHD (Fig. 3e, f). All three cases of Grade 3/4 aGvHD were of late onset (382, 455, and 528 days post HSCT; 13% at 2 years), and all these patients had received unmanipulated DLI shortly before developing aGvHD (15–40 days). One patient died of aGvHD 577 days after ATIR101 infusion, being the only death due to GvHD (death recorded as RRM because DLI was used to treat relapse). Two patients experienced aGvHD before ATIR101 infusion, apparently resulting from residual T cells contained in the CD34^+^ stem-cell graft; they did not develop GvHD after ATIR101 infusion.

### GvHD-free, relapse-free survival (GRFS)

The 12-month GRFS rate was 56.5% with TCD-haplo + ATIR101, 20% with TCD-haplo alone, 40.6% with MUD, 27.0% with MMUD, and 22.7% with UCB (Table 3). The improvement in estimated GRFS with TCD-haplo + ATIR101 was significant compared with MMUD (HR: 2.60; 95% CI: 1.24–5.47; *P* = 0.026) and UCB (HR: 3.39; 95% CI: 1.50–7.67; *P* = 0.004; Fig. 4b). Finally, estimated GRFS was not statistically significantly different between TCD-haplo + ATIR101 and MUD (HR: 1.62; 95% CI: 0.76–3.34; *P* = 1.000). The 2-year GRFS rate was 39.1% with TCD-haplo + ATIR101 (Fig. 3g). Data were similar for the ATIR101 study ITT population (Supplementary Table 1; Supplementary Figs. 2 and 5).

### Impact of donor and ATIR101 characteristics on outcome

**Fig. 5:**
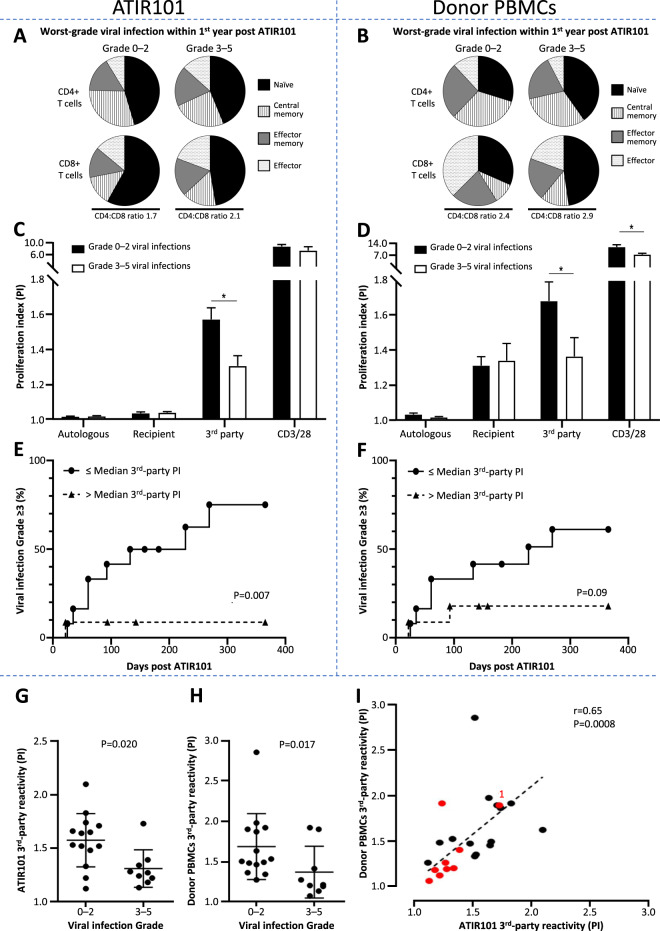
Impact of donor and ATIR101 characteristics. The characteristics of ATIR101 and donor PBMCs were evaluated for patients without viral infections or with Grade ≤2 viral infections (Grade 0–2; *n* = 14) and with Grade 3–5 viral infections (*n* = 9) within 1 year post ATIR101. **a**, **c**, **e** on the left show data using ATIR101 and **b**, **d**, **f** on the right show data using donor PBMCs. The mean proportion (%) of CD4^+^ and CD8^+^ naïve (black), central memory (striped), effector memory (grey), and effector (dots) T cells, and the average CD4:CD8 ratio, by flow cytometry is shown for ATIR101 (**a**) and donor PBMCs (**b**) according to the viral infection groups. T-cell PI of ATIR101 (**c**) or donor PBMCs (**d**) is shown for both viral infection groups, after stimulation with autologous irradiated donor cells, irradiated recipient T cells, third-party cells, and CD3/CD28 beads. Kaplan–Meier of time to Grade ≥3 viral infection for patients divided into those with “high” (>median PI; *n* = 11; circles) and “low” (≤median PI; *n* = 12; triangles) responsiveness to stimulation with third-party cells in ATIR101 (**e**) or donor PBMCs (**d**). Groups are compared using the log-rank test. Individual third-party reactivity is indicated for ATIR101 (**g**) and donor PBMCs (**h**). Correlation between T-cell PI for corresponding ATIR101 and donor PBMCs in response to stimulation with third-party cells is shown in **i** using Spearman’s correlation. Patients with Grade ≥3 viral infections are indicated in red. A patient with a Grade ≥3 viral infection with substantial third-party reactivity (in donor PBMCs and ATIR101) is indicated with a “1”. This patient was CMV positive and had a CMV-negative donor yielding a Grade 3 CMV infection. *P < 0.05. CMV cytomegalovirus, PBMC peripheral blood mononuclear cell, PI proliferation index.

The most frequent cause of NRM with TCD-haplo + ATIR101 was infection; therefore, the impact of ATIR101 characteristics on viral infections was evaluated (viral were the most frequent infections and are most likely impacted by lack of functional T cells). Patients were divided into those with Grade 3–5 viral infections (*n* = 9) and those with Grade 1/2 or no viral infection (Grade 0–2; *n* = 14) within 1 year after ATIR101. Only minor differences were found in T-cell subset distribution within ATIR101 between these groups, particularly in the CD8^+^ compartment where naïve T cells seemed increased in patients with Grade 1/2 or no viral infection (Fig. 5a).

However, functional responses against third-party antigens were significantly lower in ATIR101 cell grafts administered to patients with Grade 3–5 viral infections, compared with cell grafts received by patients with Grade 0–2 viral infections (Fig. 5c, g). Splitting third-party responsiveness of ATIR101 at the median, patients with lower third-party activity had a significantly higher risk of developing Grade ≥3 infections than those receiving cell grafts with third-party alloreactivity above the median (Fig. 5e; *P* = 0.007). To delineate the impact of the photodepletion process, the role of third-party reactivity was also evaluated in donor PBMCs. Compared with those of patients with Grade 3–5 infections, PBMCs from donors to patients with Grade 0–2 viral infections had significantly increased third-party reactivity (Fig. 5d, h). The third-party reactivity of donor PBMCs correlated with that of ATIR101 (Fig. 5i; *P* = 0.0008), so susceptibility to life-threatening viral infections was associated with a lower ability of initial donor PBMCs, and subsequently ATIR101, to respond to third-party challenges. In addition, CD8^+^ viral (EBV/CMV) dextramer-positive cells are retained in ATIR101 samples from the corresponding donor PBMCs (Table 4). IFNy release in the supernatants from re-stimulated total samples of both donor or ATIR101, also suggests that reactivity is retained in ATIR101 samples compared with corresponding donor PBMCs after co-stimulation with viral peptide pools (Table 4).

**Table 4:** CMV and EBV dextramer positive CD8^+^-enriched T cells in donor and ATIR101 samples and secreted IFNγ after re-stimulation with CMV, EBV peptide pools, or pathomix in total donor PBMC or ATIR101 samples.

Dextramers	CMV				EBV				Pathomix	
	CMV dextramer CD8^+^-enriched T cells		IFNy production upon re-stimulation with CMV peptides (pg/mL)		EBV dextramer CD8^+^-enriched T cells		IFNy production upon re-stimulation with EBV peptides (pg/mL)		IFNy production upon re-stimulation with pathomix (pg/mL)	
	Donor	ATIR101	Donor	ATIR101	Donor	ATIR101	Donor	ATIR101	Donor	ATIR101
HLA-A*0301	0	0	0	<LOD	<LOD	<LOD	19	85	>1000	65
HLA-A*0301	0.390%	0.256%	96	<LOD	<LOD	<LOD	0	<LOD	535	39
HLA-A*2402	0.058%	0.025%	#	#	0.011%	0.008%	#	#	#	#
HLA-A*2402	0	0	#	#	<LOD	<LOD	#	#	#	#
HLA-A*2402	0	<LOD	#	#	0.017%	0.013%	#	#	#	#
HLA-A*0201	<LOD	<LOD	#	#	0.012%	0.016%	#	#	#	#
HLA-A*0201	0.676%	0.289%	>1000	614	0.046%	0.019%	>1000	317	374	110
HLA-A*0201	<LOD	<LOD	#	#	1.588%	1.518%	#	#	#	#
HLA-A*2402	0	0	0	0	<LOD	<LOD	0	0	>1000	929
HLA-A*0201	<LOD	<LOD	#	#	0.061%	0.050%	#	#	#	#
HLA-A*0301	0.033%	0.037%	199	55	0.012%	<LOD	<LOD	173	>1000	>1000
HLA-A*0201	1.101%	0.434%	>1000	194	0.031%	0.015%	0	0	>1000	>1000
HLA-A*2401	0	0	0	0	<LOD	<LOD	<LOD	<LOD	137	<LOD
HLA-A*2402	0	0	<LOD	0	<LOD	<LOD	149	28	>1000	432
HLA-A*2402	0	0	#	#	<LOD	<LOD	#	#	#	#
HLA-A*0201	0.767%	0.445%	#	#	0.174%	0.075%	#	#	#	#
HLA-A*0201	0.121%	0.081%	>1000	562	0.049%	0.041%	153	50	>1000	>1000

Only donors with HLA HLA-A*0201, HLA-A*0301, and/or HLA-A*2402 were assessed for the presence of CMV- and/or EBV-specific T cells as well as the corresponding ATIR101 batch. CMV or EBV gates with <10 cells were regarded as negative and shown as <LOD. Gates without cells were set at 0. IFNy production was measured in the supernatant from total samples of donor PBMCs and ATIR101. If no IFNy was detected, the value is set at 0, samples lower than 15.6 are shown as <LOD, samples with values >1000 are set at >1000. LOD was 15.6 pg/mL and upper limit of quantification was 1000 pg/mL. # indicates IFNy ELISA invalid assay and not reported. *LOD* limit of detection.

## Discussion

The rise of haploidentical HSCT is mostly associated with the rise in T-cell-replete strategies such as PTCY [4, 5, 8]. Ease of application, low treatment costs, and general robustness, together with an unmet need in patients without a suitable, timely available HLA-matched donor, have certainly contributed to this impressive development [2, 8]. However, the PTCY approach, such as allogeneic HSCT in general, is challenged by the appearance of residual GvHD and relapse and still requires posttransplant immunosuppression [3, 15, 16, 18, 19]. Immunosuppression is associated with an infection risk, as well as nephrotoxicity and metabolic, neurologic, and hepatic complications, meaning patients must be closely monitored and may need to discontinue these drugs due to toxicity [40, 41]. ATIR101 is an adjunctive cellular therapy, selectively depleted of recipient-alloreactive T cells, which is administered after TCD-haplo to provide anti-infective and anti-leukemic activity without the use of posttransplant immunosuppression. In the absence of a defined standard approach to haploidentical HSCT, an observational registry study was undertaken to provide control groups matching the inclusion/exclusion criteria of the ATIR101 study to compare outcomes with classical TCD-haplo and estimate the benefit of ATIR101, as well as to compare with other standard-of-care approaches using MUD, MMUD, and UCB as donor source. TCD-haplo + ATIR101 was well tolerated, improved outcomes versus TCD-haplo alone, and resulted in 1-year GRFS that was not significantly different to MUD. As value-based healthcare becomes a priority, composite endpoints such as GRFS reflecting disease status and quality of life are gaining importance in HSCT [37]. It is important to note that results for the control study groups reported in the present study are in line with those from the CIBMTR with 1-year survival for malignant diseases receiving MUD of 68% (versus 85.9% in this study), 60% for MMUD (versus 64.9% in this study), and 59% for UCB (versus 54.5% in this study), confirming the validity of the comparisons [42]. Therefore, the results presented here demonstrate the potential benefit of ATIR101 to the entire field of allogeneic HSCT.

The absence of Grade 3/4 aGvHD in the first year after TCD-haplo + ATIR101 is compelling in view of delivery of a high dose of HLA-mismatched donor lymphocytes and the lack of posttransplant immunosuppression. All aGvHD events in the first year were Grade 1/2, with two out of nine patients having GvHD only from the CD34^+^-graft before ATIR101 infusion. By comparison, in a meta-analysis of haploidentical HSCT plus PTCY, the 100-day rate of Grade 3/4 aGvHD ranged from 0 to 25% [43]. There, 2-year rates of moderate/severe cGvHD ranged from 0 to 36% [43], whereas only one patient with TCD-haplo + ATIR101 experienced moderate/severe cGvHD, in line with Phase 1 data [34], demonstrating that ex vivo photodepletion successfully removes GvHD-causing cells.

ATIR101 serves as an adjunctive to TCD-haplo, a historical standard of haploidentical HSCT [44, 45]. Despite the groundbreaking achievement of engraftment across major HLA disparities without high-grade GvHD, overall outcomes with TCD-haplo were disappointing, with very high NRM and relapse rates [20]. Indeed, infection was reported to be the cause of ~60–70% of NRM with TCD-haplo [20, 46]. Considering these challenges, NRM at 6 months was chosen as primary endpoint of the ATIR101 study, which was only 13%. Also, 1-year NRM was significantly lower than with TCD-haplo alone. Although 21.7% of NRM at 1 year were infection related in the ATIR101 study, this still compares favorably versus 40.0% infection-related NRM with TCD-haplo alone, suggesting ATIR101 has anti-infection activity that could help provide immune protection after TCD-haplo or following other transplant protocols. The rate of NRM at 2 years was 43.5%, with eight NRM being infection related; however, one patient who was not adequately screened for adenovirus subsequently died of adenovirus infection and another developed fatal JC virus encephalopathy potentially as a result of multiple doses of rituximab and cyclophosphamide treatment for posttransplant lymphoproliferative disorder. Therefore, more rigorous infection screening, along with the addition of ATIR101, is important for the improvement of NRM following TCD-haplo. In addition, the use of unmanipulated DLI in 4/10 patients with NRM in the ATIR101 study may also have contributed to NRM rate, emphasizing the need for development of manipulated DLIs, such as ATIR101 [47].

In this study, ATIR101 third-party reactivity was associated with the occurrence of fewer clinically relevant viral infections, suggesting increased T-cell fitness leading to superior antiviral immunity. To our knowledge, this is the first study to highlight a possible role for strength of such donor-cell immune reactivity on post-HSCT infection control. Importantly, these data also identified a potential donor-selection tool considering most patients have multiple haploidentical donors available [48], and the optimal donor could be selected before cell collection by measuring third-party reactivity in donor PBMCs. If only one donor is available, or third-party reactivity is limited in all donors, donor vaccination before apheresis and adoptive transfer of antivirus-specific T cells may be considered [49–51]. Our findings regarding the contribution of donor T cells to HSCT outcomes are also in line with donor T-cell genomic profile predicting for GvHD after HLA-matched T-cell-replete transplants and warrant further investigation [52]. Although alloreactive responses have been attributed to naïve T cells [53–55], photodepletion led to a relative enrichment of naïve cells while effectively diminishing recipient alloreactivity, indicating that patient-reactive naïve T cells can be selectively depleted. Having greater diversity than memory cells [56], remaining naïve T cells could provide protection against infectious agents not previously encountered by the donor. In fact, patients with low-grade or no viral infections seemed to have more CD8^+^ naïve cells in ATIR101 than those with Grade ≥3 viral infections. The role of naïve cells in protection against viral infections may represent an important consideration in the favorable outcome observed.

In haploidentical HSCT using PTCY, high relapse rates have been described as a potential consequence of depletion of graft-versus-leukemia responses and use of posttransplant immunosuppression [18, 57–59]. The maintenance of graft-versus-leukemia effects of ATIR101 is supported by the lower relapse rate (8.7%) versus TCD-haplo alone (20.0%). Beyond classical TCD-haplo, ATIR101 could even be a beneficial adjunctive to alpha–beta T-cell-CD19-depleted haploidentical HSCT, providing additional graft-versus-infection and -leukemia effects to the immunity of NK cells and gamma–delta T cells [21, 22, 60–62].

Results presented suggest that TCD-haplo + ATIR101 may represent a promising alternative to other approaches such as the applied standard of PTCY and MUD HSCT. In view of the promising Phase II data presented here, and despite the absence of an applied standard for haploidentical HSCT, a large, multicenter, Phase 3 randomized trial was initiated, which was designed to show superiority in GRFS of TCD-haplo using CD34^+^ selection and the addition of ATIR101 over T-cell-replete haploidentical HSCT using PTCY (NCT02999854). This trial was terminated prematurely based on Sponsor’s decision.

The authors understand the overall difficulty of performing a randomized trial with an advanced therapy medicinal product (ATMP) with regard to a number of factors, including the diversity of T-replete transplant procedures in the control arm, sophistication of production, and economical resources required. Use of ATIR101 and other ATMPs are clearly more technologically demanding and costly than the PTCY approach. The regulatory pathway for cellular and gene therapy ATMPs as “drugs” is challenging, and resolution of such crucial issues associated with clinical trial design and performance is needed for improved patient quality of life, translation into standard of care, and economic endorsement.

## Supplementary information

Supplementary Materials
