# Supplementary material for: ATIR101 administered after T-cell-depleted haploidentical HSCT reduces NRM and improves overall survival in acute leukemia
Source: Leukemia. 2020 Feb 11;34(7):1907–23. doi: 10.1038/s41375-020-0733-0 (PMC7326707; doi:10.1038/s41375-020-0733-0)
Supplement: Supplementary file 1 — Supplementary Materials [file 41375_2020_733_MOESM1_ESM.docx]

**Supplementary appendix**

**Supplementary methods:**

**Informed consent and ethics**

Informed consent was obtained from patients and donors in the ATIR101 study, and all treatment was administered under protocols approved by appropriate independent ethics committees at participating study centers. Both the ATIR101 study and control study were conducted in accordance with the ethical principles of the Declaration of Helsinki, which are consistent with the International Conference on Harmonization Guideline for Good Clinical Practice as well as the applicable regulatory requirements.

**Control study: key inclusion and exclusion criteria**

Inclusion criteria included: patients between 18 and 65 years of age with acute myeloid leukemia in remission at the time of the transplantation, acute lymphoblastic leukemia in remission at the time of the transplantation, or myelodysplastic syndrome. Exclusion criteria included: stem-cell transplantation prior to the transplantation qualifying for the study.

**ATIR101 study: key inclusion and exclusion criteria**

Inclusion criteria included: patients between 18 and 65 years of age who were eligible for haploidentical hematopoietic stem-cell transplantation (HSCT; according to the investigator) with acute myeloid leukemia in first remission with high-risk features or in second or higher remission; acute lymphoblastic leukemia in first remission with high-risk features or in second or higher remission; or myelodysplastic syndrome that is transfusion dependent or intermediate or higher IPSS-R risk group. Exclusion criteria included: availability of a suitable matched related or unrelated donor following a donor search, prior stem-cell transplantation, in second or higher remission with the previous remission having lasted <6 months, and estimated probability of surviving less than 3 months.

**ATIR101 study: Determination of sample size**

This sample size is based on Simon’s two-stage minimax phase II design.^1^ The following assumptions were made: The development of ATIR101 should be reconsidered if the NRM rate at 6 months is 45% or greater; further development of ATIR101 is of definite interest if the NRM rate at 6 months is 15% or lower; the statistical hypotheses of interest are H_0_: r≥45% against HA: r≤15%, where r is the

NRM rate at 6 months; the type I error rate (α, probability of accepting a treatment with a high TRM rate at 6 months, a false positive outcome) is set to at most 5%; The type II error rate (β, probability of rejecting a treatment with a low TRM rate at 6 months, a false negative outcome) is set to at most 5%.

**ATIR101 production under good manufacturing practice**

ATIR101 batches were produced under good manufacturing practice conditions at DRK-Blutspendedienst Baden-Württemberg-Hessen (Frankfurt am Main, Germany) or Centre d’Excellence en Thérapie Cellulaire (Hôpital Maisonneuve-Rosemont, Montréal, Canada). Quality control, including T-cell viability and specificity, was performed at the Kiadis Laboratory (Amsterdam, the Netherlands). Final cellular product safety tests, including sterility, endotoxin levels, and mycoplasma testing assays, were performed at MicroSafe/Eurofines Laboratories (Leiden, the Netherlands).

Donors and patients underwent mononuclear cell-enriched leukapheresis to obtain cells and plasma for a mixed lymphocyte reaction. Donor plasma was collected during leukapheresis, filtered with a 0.22 µm filter, and heat-inactivated at 56 °C for 60 mins.

Leukapheresis-obtained cells were washed and processed by automated mononuclear cell purification using a Sepax device (Biosafe, Eysins, Switzerland), following the standardized Sepax NeatCell protocol. Subsequently, donor peripheral blood mononuclear cells (PBMCs; 1×10^6^/mL) were co-cultured with gamma-irradiated (50 Gy) recipient PBMCs 1:1 in X-VIVO 15™ medium (Lonza, Basel, Switzerland), containing 2% donor plasma and 50 IU/mL interleukin-2 (proleukin, Novartis), for 4 days under static conditions.

After the mixed lymphocyte reaction, cells were centrifuged and pooled in X-VIVO 15™ containing 2.5% donor plasma and 5 µM TH9402 (Kiadis Pharma, Amsterdam) and incubated at 37 °C, 5% CO_2_. After 40 mins of coloration, cells were centrifuged (500 g, 15 mins) to remove coloration medium. The cell pellet was resuspended in X-VIVO 15™ with 10% donor plasma and incubated at 37 °C, 5% CO_2_ for 90 mins to allow extrusion of the dye. After extrusion, cells were immediately placed on the photodynamic therapy device (Kiadis Pharma, Amsterdam) and exposed to visible light until an energy dose of 5 J/cm^2^ was delivered. Cells were then collected and centrifuged (500 g, 15 mins) and the percentage of viable T cells in the total cell population was determined using flow cytometry. The cell suspension volume required to formulate the product and test and retain samples (2×10^6^ cells/kg bodyweight) was withdrawn and centrifuged (500 g, 15 mins); cells were resuspended in saline with 20% donor plasma. From this suspension, 10 mL was transferred to the final product bag. Next, 10 mL pre-cooled (2–8 °C) dimethyl sulfoxide (DMSO)-containing medium with 40% donor plasma and 20% DMSO was added to the suspension in the final product bag, and the product was frozen until administered to the patient. Immediately thereafter, DMSO-containing medium was added 1:1 to the remaining suspension and frozen, formulating samples required for further quality control and characterization assays.

**Cell composition**

Donor PBMCs (starting material) and ATIR101 (final product) were phenotyped for presence of T cells (CD3^+^), monocytes (CD14^+^), B cells (CD19^+^), and natural killer (NK) cells (CD3^-^/CD16^+^+CD56^+^). The monoclonal antibodies used were: anti-CD45-V450 (HI30, 560367), anti-CD3-V500 (UCHT1, 561416), anti-CD19-FITC (HIB19, 555412), anti-CD14-APC-Cy7 (MϕP9, 557831), anti-CD16-PE (3G8, 555407), anti-CD56-PE (B159, 555516) – all from BD Biosciences, Breda, the Netherlands – and anti-TCRαβ-PE-Cy7 (IP26, 306720) and anti-TCRγδ-APC (B1, 331212) – both from Biolegend, San Diego, CA, USA. Viability was assessed by 7-AAD labeling on thawed ATIR101 batches and specifications set post thaw. Briefly, specific antibodies were added to 2.5×10^5^ cells/well and incubated for 15 mins in the dark at room temperature (RT). After washing, 5 µL of 7-amino-actinomycin D was added and the cells were incubated for at least 5 mins in the dark at RT. Cell composition was then determined by multiparameter flow cytometry (BD FACSVerse) and processed using BD FACSuite software.

**Immunophenotypic analysis of T cells of ATIR101 and donor PBMCs**

Surface expression of T-cell antigens was evaluated by direct immunofluorescence as described for cell composition above. The monoclonal antibodies used were: anti-CD3-V500 (UCHT1, 561416), anti-CD4-APC-H7 (RPA-T4, 560158), anti-CD8-PE-Cy7 (RPA-T8, 557746), anti-CD45RO-BV421 (UCHL1, 562641) and anti-CD62L-PE (DREG-56, 555544, all from BD Biosciences, Breda. Samples were all tested in triplicate (if not enough cells were available, the number of replicates was reduced).

**Carboxyfluorescein succinimidyl ester (CFSE)-dilution-based proliferation assay**

A CFSE-dilution-based proliferation assay was used to determine the specificity of depletion of alloreactive T cells and the potency of remaining cells. Briefly, 2 µM CFDA-SE (Invitrogen Life Technologies, Bleiswijk, the Netherlands) was added to 2×10^6^ viable T cells/mL. The cells were incubated at 37 °C for 7 mins in the dark before addition of cold X-VIVO 15™ with 10% plasma and incubated at 2–8 °C for 5 mins in the dark to quench the labeling. Cells were then washed three times by centrifuging at 500 g for 7 mins at RT. Next, the cells were resuspended in CFSE culture medium (X-VIVO 15™ with 10% plasma and 1 U/mL interleukin-2) to a concentration of 1×10^6^ viable cells/mL.

Co-cultures were set up using 1×10^5^ cells/well at 1:1 labeled T-cell:stimulus ratio. Four different proliferation-inducing stimuli were used: irradiated autologous donor cells, irradiated recipient cells, irradiated third-party cells (mix from 5 random individuals), and anti-CD3/CD28 beads. Cells were incubated at 37 °C, 5% CO_2_ for 5 days; the proliferation pattern was then determined using Modfit LT-analysis (VSH Software).

**Dextramer staining for CD8^+^ viral-positive cells (EBV/CMV)**

Cells were thawed and CD8^+^ cells enriched by negative selection using a MACS-based approach. Dextramers were used for identification of the CMV or EBV specific T-cells in donor PBMCs and ATIR101 using the following dextramers (Immudex) to common epitopes: HLA-A*0201/GLCTLVAML (EBV/BMLF1), HLA-A*0301/KLGGALQAK (CMV-IE1), HLA-A*0301/RLRAEAQVK (EBV/EMNA3A), HLA-A*2402/QYDPVAALF (CMVpp65), HLA-A*2402/TYGPVFMCL (EBV-LMP2), HLA-A*0201/NLVPMVATV (CMV/pp65). Only donors with HLA HLA-A*0201, HLA-A*0301 and/or HLA-A*2402 were assessed for the presence of CMV and/or EBV specific T-cells as well as the corresponding ATIR101 batch. Cells were stained and dextramer positive cells were assessed as a percentage of live (7-AAD negative), CD45^+^, CD3^+^, CD4^-^, CD8^+^ cells. CMV or EBV gates with <10 cells were regarded as negative and shown as below the limit of detection. Gates without cells were set at 0.

**IFNy measurement secreted in supernatant of restimulated cells**

IFNy production upon stimulation by CMV, EBV or pathomix was assessed by a standard ELISA (LEGEND Max, Biolegend). In short, cells were thawed and responder cells (complete donor or ATIR101 samples), were co-cultured with donor cells, which served as antigen presenting cells for 5 days, together with the viral peptivator mixes (Miltenyi Biotech) or toxoid at 0.25 ug/mL or at 10 ug/mL for the lysate (CMV [peptivator pp65 and IE-1 mixture], EBV [peptivator EBV] or pathomix [tetanus toxoid, aspergillus fumigates lysate and peptivator Candida Albicans MP65]). Supernatant was harvested and human IFNy ELISA was performed. The amount of IFNy is presented from valid assays, samples were not subjected to repeat analysis. Limit of detection was 15.6 pg/mL and upper limit of quatification was 1000 pg/mL.

**Patient conditioning and HSCT in the ATIR101 study**

Patients received one of two myeloablative conditioning regimens: (1) total body irradiation (TBI) regimen, which consisted of fractionated TBI (200 cGy twice daily for 3 days on Days –10 to –8 [1200 cGy in 6 fractions]) followed by fludarabine (30 mg/m^2^ intravenously [i.v.] once daily for 5 days on Days –7 to –3) and thiotepa (5 mg/kg i.v. twice daily for 1 day on Day –7); or (2) non-TBI regimen, which consisted of fludarabine (30 mg/m^2^ i.v. once daily for 5 days on Days –8 to –4), thiotepa (5 mg/kg i.v. twice daily for 1 day on Day –7), and melphalan (60 mg/m^2^ i.v. once daily for 2 days on Days –2 and –1). In addition, all patients received anti-thymocyte globulin (Thymoglobulin®, Sanofi Genzyme, Cambridge, MA, USA) 2.5 mg/kg once daily for 4 days on Days –5 to –2, as a continuous i.v. infusion for 8 hours. During the course of anti-thymocyte globulin, patients received methylprednisolone (2 mg/kg/day i.v.).

The collection and preparation of the donor stem-cell graft was performed according to institutional procedures at the study center. Peripheral blood stem cells from the donor were mobilized with granulocyte colony-stimulating factor administered subcutaneously at a dose of approximately 8 μg/kg twice daily for approximately 4–7 days. The peripheral blood stem cells were collected by apheresis. To ensure a consistently highly purified stem-cell graft, clinical sites used the CliniMACS® CD34 isolation system (Miltenyi Biotec, Bisley, UK) as part of their institutional procedures for preparing the stem-cell graft. According to the Perugia protocol for haploidentical transplants, the CD34-selected stem-cell graft was targeted to contain at least 5×10^6^ CD34^+^ cells/kg but if possible 8–11×10^6^ CD34^+^ cells/kg with a maximum of 3×10^4^ CD3^+^ cells/kg as assessed by flow cytometry.

**Information collected in the control study**

The following information was to be collected at baseline: patient demographics (date of birth, sex), hematologic disease (malignancy classification, date of diagnosis, disease status, number of previous remissions), and bone marrow biopsy/aspirate (date, biopsy and/or aspirate cellularity, % myeloblasts). The following information was to be collected regarding transplantation: conditioning regimen (myeloablative/reduced intensity), TBI (none/fractionated/non-fractionated), chemotherapy, serotherapy, donor characteristics (date of birth, sex, relationship to patient, human leukocyte antigen (HLA) compatibility by HLA type [HLA-A, -B, -C, -DR, -DQ where available], number of HLA mismatches between umbilical cord blood (UCB) units [HLA-A, -B, -DR]), HSCT/UCB transplantation date, dose of viable CD34^+^ and CD3^+^ cells/kg, and engraftment (first days of occurrence of neutrophil and platelet engraftment criteria). The following was collected during continuous follow-up until 12 months after transplantation: disease relapse/progression (reported term, start date, outcome, stop date, method of evaluation, action taken), graft-versus-host disease (GvHD; type, start date, outcome, stop date, maximum severity, action taken), infections and veno-occlusive disease leading to hospitalization (Epstein-Barr virus [EBV] infection or reactivation, post-transplant lymphoproliferative disorder, cytomegalovirus infection or reactivation, adenovirus infection, JC or BK virus infection, veno-occlusive disease, fungal infections), and mortality (date of death, cause of death – specification and investigator classification [disease relapse/disease progression/non-relapse mortality (NRM)/other known cause unrelated to the transplantation procedure or underlying disease, e.g. accident, suicide) and in case of NRM also NRM class (GvHD/infection/graft failure-graft rejection/other). Finally, the following was recorded at last follow-up (12 months ± 1 month after transplantation): biochemistry (serum creatinine, bilirubin, alanine aminotransferase, aspartate aminotransferase, pulmonary function test (total lung capacity, lung obstruction, diffusion capacity), and echocardiogram or multigated acquisition scan.

**Endpoints**

In the ATIR101 study, overall survival (OS) was defined as the time from HSCT until death from any cause; relapse-related mortality (RRM) was defined as death due to disease relapse or disease progression; progression-free survival (PFS) was defined as the time from HSCT until relapse, disease progression, or death, whichever occurred first.

Patient samples were analyzed using flow cytometry on site to assess recovery of total lymphocytes (CD3^+^), helper T cells (CD3^+^/CD4^+^), cytotoxic T cells (CD3^+^/CD8^+^), B cells (CD19^+^), and NK cells (CD3^–^/CD56^+^). Assessment time points were defined relative to ATIR101 administration in the earlier study period (Weeks 1–8) and relative to HSCT in the later study period (Months 4–24) to optimally capture the more-frequent follow-up of patients early after ATIR101 infusion.

Infections were defined as a clinically apparent infectious disease with symptoms or a viral reactivation. National Cancer Institute Common Terminology Criteria for Adverse Events version 4.0 was used to grade infection severity whereby: Grade 3 is with significant symptoms requiring hospitalization or invasive intervention, transfusion, elective interventional radiologic procedure, therapeutic endoscopy or operation; Grade 4 is life threatening or disabling; Grade 5 is death related to the adverse event.

Patients were monitored for cytomegalovirus and EBV using quantitative polymerase chain reaction, at least weekly until Week 8 after ATIR101 infusion, monthly until Month 6 after the HSCT, and every 2 months until Month 12 after the HSCT. Cytomegalovirus monitoring was conducted for all patients, and EBV monitoring was conducted in the case of an EBV-positive patient or donor or as indicated. GvHD was graded according to standard criteria.^2, 3^

Endpoints for the observational control study were NRM, RRM, OS, PFS, and the incidence and severity of GvHD. The primary comparison between the ATIR101 and control studies was with the T-cell-depleted haploidentical (TCD-haplo) HSCT group without ATIR101. Secondary comparisons included validation of the effects of ATIR101 against matched-unrelated donor and comparability of TCD-haplo + ATIR101 with matched-unrelated donor, mismatched-unrelated donor, and double UCB. The primary endpoint for the comparison was 6-month NRM and secondary endpoints included 12-month NRM and 6- and 12-month OS as well as RRM, PFS, and the incidence and severity of GvHD.

An exploratory analysis was conducted on the composite endpoint of GvHD-free, relapse-free survival (GRFS), which was defined as time from randomization until Grade 3/4 acute GvHD, chronic GvHD requiring systemic immunosuppressive treatment, disease relapse, or death, whichever occurs first.^4^

**Supplementary results:**

**HSCT and ATIR101**

The median time between apheresis (to collect lymphocytes for ATIR101) and HSCT was 29 days (range 13–64). Patients in the TCD-haplo + ATIR101 population received the planned ATIR101 dose of 2.0×10^6^ viable T cells/kg and were infused at a median of 28 days (range 28–73) post HSCT after confirmation of engraftment (Figure 1A), except for one patient who received ATIR101 before neutrophil engraftment had been demonstrated and one patient who received ATIR101 before platelet engraftment had been demonstrated. In two patients, ATIR101 infusion was delayed beyond 32 days post HSCT (73 days and 39 days) because the patients had developed GvHD. After resolution of GvHD, these patients were infused with the intended dose of ATIR101. No graft failures were observed in the TCD-haplo + ATIR101 population.

**Immune reconstitution in the TCD-haplo + ATIR101 population**

Lymphocyte numbers gradually increased from Month 4 after HSCT (Month 3 after ATIR101 infusion; Figure 2). Similarly, CD3^+^, CD4^+^, and CD8^+^ T-cell counts increased from Month 4 onwards. CD19^+^ B-cell counts increased early in the first 2–3 weeks after HSCT and mean CD56^+^ NK count increased relative to levels before HSCT by Week 4, which remained consistent for the remainder of the study visits.

**Supplementary figure legends:**

**SF1. CONSORT diagrams for the ATIR101 study and observational control study**A) ATIR101 study (CR-AIR-007). Of 31 patients enrolled in the ATIR101 study, 26 patients received HSCT (ITT population). For one patient, ATIR101 could not be manufactured because of the high red blood cell count in the patient’s apheresis, and four patients were discontinued at this stage due to rejection of the ATIR101 batch and/or relapse before start of the conditioning regimen (*one patient in the ATIR101 study was discontinued due to relapse and rejection of ATIR101 batch). After HSCT, three patients were discontinued due to death, graft failure, and rejection of the ATIR101 batch, respectively. Therefore, twenty-three patients received ATIR101 (TCD-haplo + ATIR101).

B) Control study (CRI-AIR-006). All eligible patients were included in the study, except for 183 MUD patients (selected at random because the protocol limited the number of patients per arm).
HSCT, hematopoietic stem-cell transplantation; ITT, intention-to-treat; MMUD, mismatched unrelated donor; MUD, matched unrelated donor; TCD-haplo, T-cell-depleted haploidentical HSCT; UCB, double umbilical cord blood.

**SF2. Outcomes in the ATIR101 study ITT population (N=26)**

Kaplan–Meier plots are shown for OS (A), PFS (B), and GRFS (G). Cumulative incidence plots taking into account competing risks are shown for RRM (C), NRM (D), acute GvHD Grade 2–4 and Grade 3–4 (E), and moderate/severe chronic GvHD (F).

GRFS, GvHD-free, relapse-free survival; GvHD, graft-versus-host disease; HSCT, hematopoietic stem-cell transplantation; ITT, intention-to-treat; NRM, non-relapse mortality; OS, overall survival; PFS, progression-free survival; RRM, relapse-related mortality.

**SF3.** **Outcomes in the TCD-haplo + ATIR101 population (N=23) and the control study TCD-haplo (N=35), MUD (N=64), MMUD (N=37), and UCB (N=22) populations**

Kaplan–Meier plots are shown for OS (A), and PFS (B). Plots of cumulative incidence taking into account competing risk are shown for RRM (C), chronic GvHD (D), acute GvHD Grade 2–4 (E), and acute GvHD Grade 3–4 (F). Plots show patients receiving TCD-haplo + ATIR101 (pink) and patients from the control study who received TCD-haplo (orange), MUD (brown), MMUD (green), or double UCB (blue). For (A) and (B), HRs and their corresponding 95% CIs are presented, and groups are compared using the log-rank test with Bonferroni correction for multiple comparisons. For (C) to (F), groups were compared using Gray’s test.

CI, confidence interval; GvHD, graft-versus-host disease; HR, hazard ratio; HSCT, hematopoietic stem-cell transplantation; MMUD, mismatched-unrelated donor; MUD, matched-unrelated donor; OS, overall survival; PFS, progression-free survival; RRM, relapse-related mortality; TCD-haplo, T-cell-depleted haploidentical HSCT; UCB, umbilical cord blood.

**SF4.** **Outcomes in the ATIR101 ITT population (N=26) and the control study TCD-haplo (N=35), MUD (N=64), MMUD (N=37), and UCB (N=22) populations**

Kaplan–Meier plots are shown for OS (A) and PFS (B). Plots of cumulative incidence taking into account competing risk are shown for RRM (C), chronic GvHD (D), acute GvHD Grade 2–4 (E), and acute GvHD Grade 3–4 (F). Plots show the ATIR101 study ITT population (pink) and patients from the control study who received TCD-haplo (orange), MUD (brown), MMUD (green), or double UCB (blue). For (A) and (B), HRs and their corresponding 95% CIs are presented, and groups are compared using the log-rank test with Bonferroni correction for multiple comparisons. For (C) to (F), groups were compared using Gray’s test.

CI, confidence interval; GvHD, graft-versus-host disease; HR, hazard ratio; HSCT, hematopoietic stem-cell transplantation; ITT, intention-to-treat; MMUD, mismatched-unrelated donor; MUD, matched-unrelated donor; OS, overall survival; PFS, progression-free survival; RRM, relapse-related mortality; TCD-haplo, T-cell-depleted haploidentical HSCT; UCB, umbilical cord blood.

**SF5.** **Cumulative incidence of NRM and Kaplan–Meier of GRFS for the ATIR101 study ITT population (N=26) and the control study TCD-haplo (N=35), MUD (N=64), MMUD (N=37), and UCB (N=22) populations**

A) Cumulative incidence plot of NRM taking into account competing risk and B) Kaplan–Meier of GRFS over 1 year for the ATIR101 study ITT population (pink) and patients from the control study who received TCD-haplo (orange), MUD (brown), MMUD (green), or double UCB (blue). For (A), groups were compared using Gray’s test. For (B), HRs and their corresponding 95% CIs are presented, and groups are compared using the log-rank test with Bonferroni correction for multiple comparisons.

CI, confidence interval; GRFS, graft-versus-host disease-free, relapse-free survival; HR, hazard ratio; HSCT, hematopoietic stem-cell transplantation; ITT, intention-to-treat; MMUD, mismatched-unrelated donor; MUD, matched-unrelated donor; NRM, non-relapse mortality; TCD-haplo, T-cell-depleted haploidentical HSCT; UCB, umbilical cord blood.

**Supplementary figures**


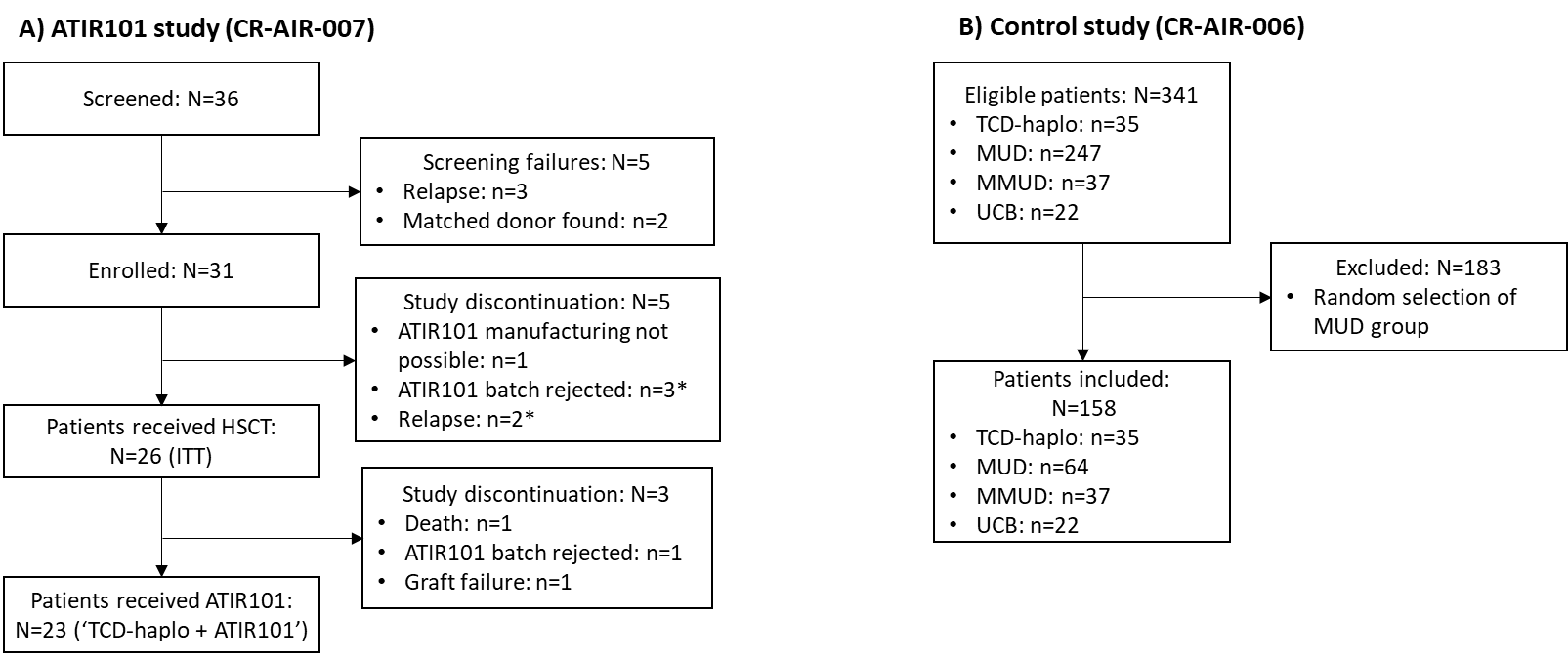
SF1. CONSORT diagrams for the ATIR101 study and observational control study

**
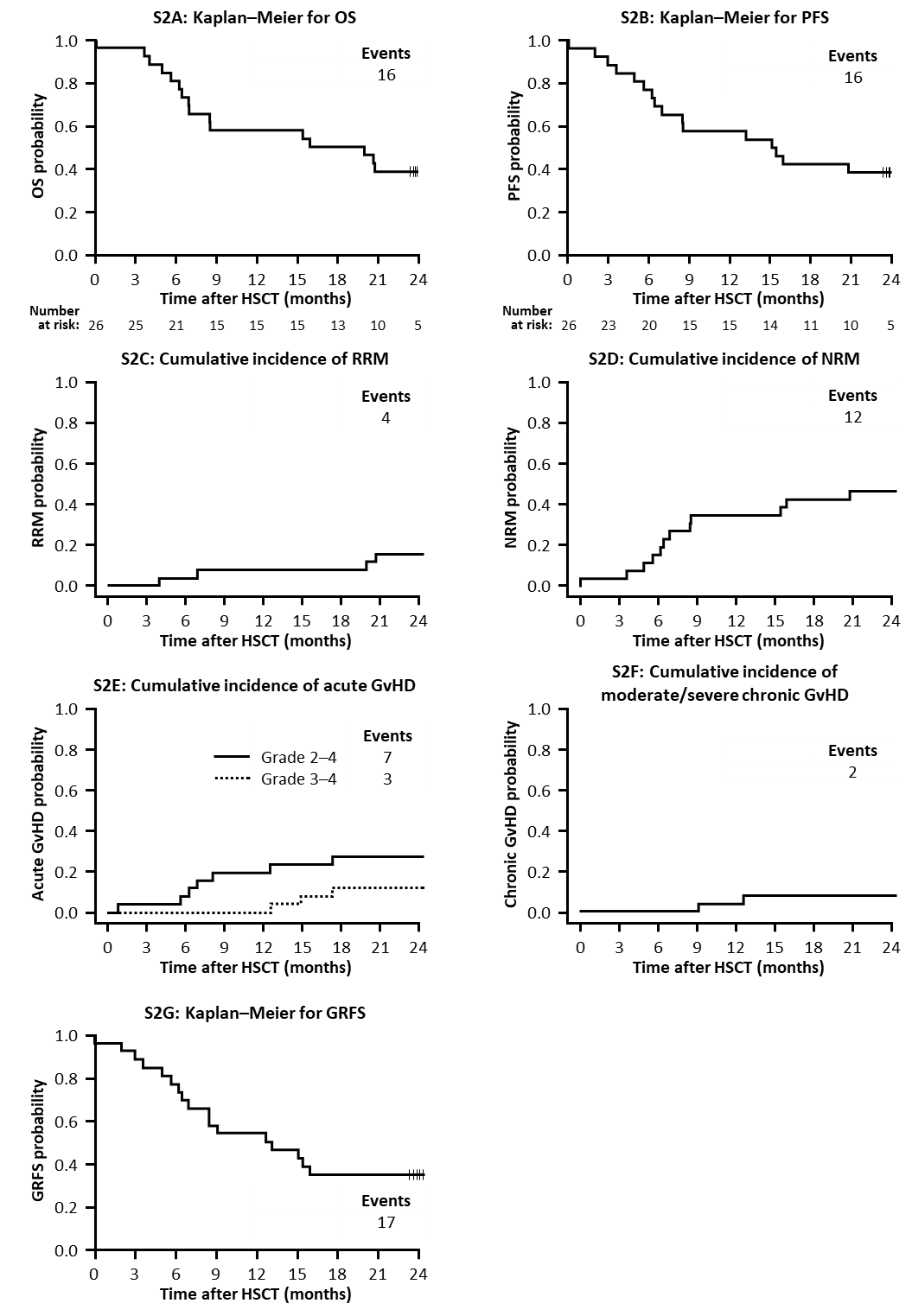
SF2. Outcomes in the ATIR101 study ITT population (N=26)**

**
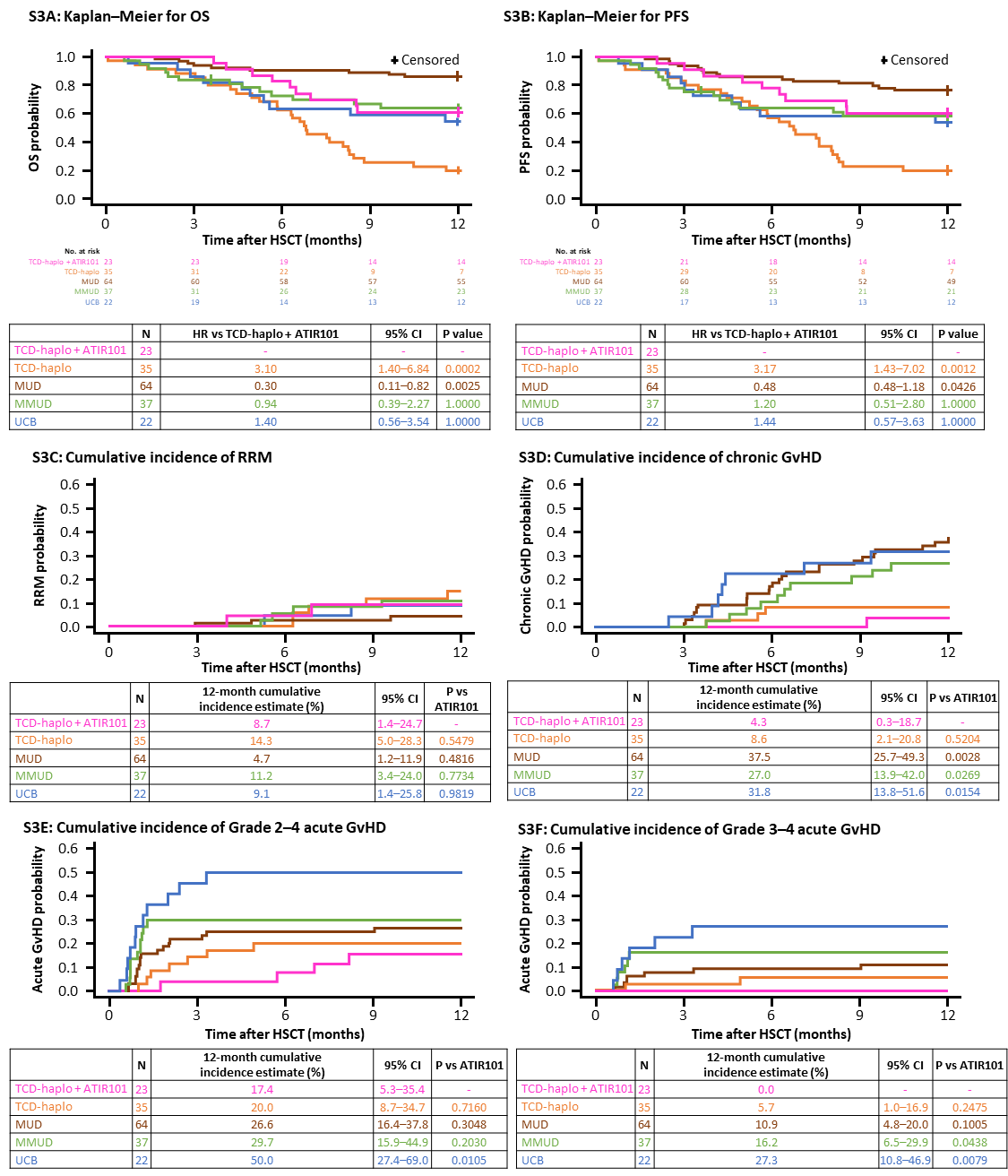
SF3: Outcomes in TCD-haplo + ATIR101 population (N=23) and the control study TCD-haplo (N=35), MUD (N=64), MMUD (N=37), and UCB (N=22) populations**

**
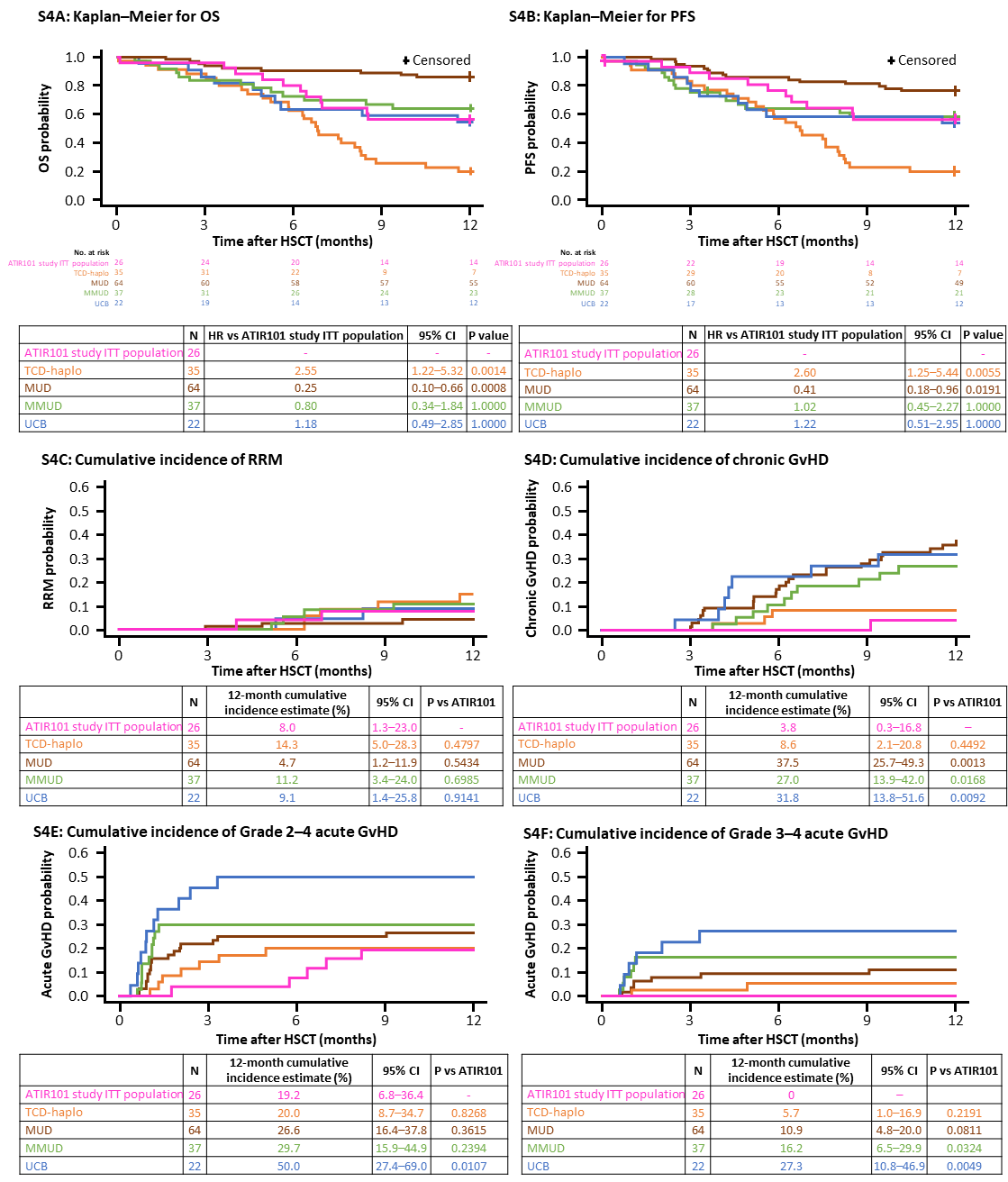
SF4: Outcomes for the ATIR101 ITT population (N=26) and the control study TCD-haplo (N=35), MUD (N=64), MMUD (N=37), and UCB (N=22) populations**

**
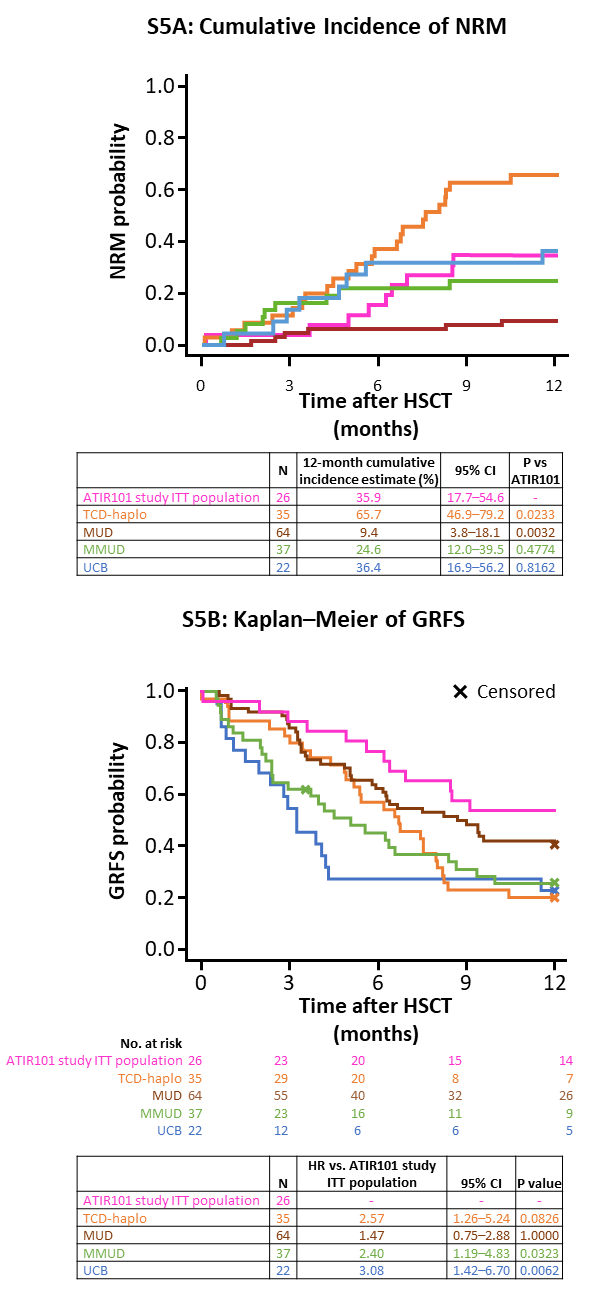
SF5. Cumulative incidence of NRM and Kaplan–Meier Estimate of GRFS in the ATIR101 Study ITT Population (N=26) and the control study TCD-haplo (N=35), MUD (N=64), MMUD (N=37), and UCB (N=22) populations**

**Supplementary Tables**

**ST1. Survival, NRM, GvHD, relapse, and PFS in the ATIR101 study ITT population (point estimates)**

|  | **ATIR101 study: ITT  (N=26)** |
| --- | --- |
| **Patients with NRM event, n (%)**  6 months  12 months  24 months | 4 (15.4)  9 (34.6)  12 (46.2) |
| **Grade 2–4 acute GvHD (cumulative), n (%)***  6 months  12 months  24 months | 2 (7.7)  5 (19.2)  7 (26.9) |
| **Grade 3–4 acute GvHD (cumulative), n (%)***  6 months  12 months  24 months | 0 (0.0)  0 (0.0)  3 (11.5) |
| **Chronic GvHD (cumulative), n (%)***  6 months  12 months  24 months | 0 (0.0)  1 (3.8)  2 (7.7) |
| **Relapse (cumulative), n (%)**  6 months  12 months  24 months | 2 (7.7)  2 (7.7)  4 (15.4) |
| **Patients with RRM event,^†^ n (%)**  6 months  12 months  24 months | 1 (3.8)  2 (7.7)  4 (15.4) |
| **PFS, n (%)**  6 months  12 months  24 months | 20 (76.9)  15 (57.7)  10 (38.5) |
| **GRFS, n (%)**  6 months  12 months  24 months | 20 (76.9)  14 (53.8)  9 (34.6) |
| **OS, n (%)**  6 months  12 months  24 months | 21 (80.8)  15 (57.7)  10 (38.5) |
| * Each patient is represented with the maximum severity; ^†^ As assessed by investigator.  GRFS, GvHD-free, relapse-free survival (acute GvHD Grade 3–4, chronic GvHD requiring systemic use of immunosuppressive medication, relapse, or death, whichever comes first in the first post-HSCT year); GvHD, graft-versus-host disease; HSCT, hematopoietic stem-cell transplantation; ITT, intention-to-treat; NRM, non-relapse mortality; OS, overall survival; PFS, progression free survival; RRM, relapse-related mortality. | |

**ST2. Use of unmanipulated DLI and cause of NRM in the TCD-haplo + ATIR101 study**

| **Timing post HSCT** | **n** | **Unmanipulated DLI (Days post HSCT)** | **Indication  for DLI** | **Days from  DLI to NRM** | **Cause of NRM** | **Details** |
| --- | --- | --- | --- | --- | --- | --- |
| **<6 months** | 3 | Yes (146) | JC virus encephalopathy | 4 | Infection (viral) | - JC virus encephalopathy |
|  |  | Yes (81/103) | Adenovirus infection | 7/29 | Infection (viral) | - Disseminated adenovirus |
|  |  | No | - | - | Other | - Presumed pulmonary embolism |
| **6–12 months** | 4 | No | - | - | Infection (fungal) | - Pulmonary Infection |
|  |  | No | - | - | Infection (unknown) | - Acute respiratory distress syndrome |
|  |  | No | - | - | Other | - Multiple organ failure due to infectious and auto immune complications |
|  |  | No | - | - | Infection (fungal) | - Respiratory infection |
| **>12 months** | 3 | Yes (342) | Low lymphocytes | 127 | Infection (unknown) | - GVHD post-DLI requiring intensive immune suppression - Septic shock |
|  |  | No | - | - | Infection (bacterial) | - Sepsis |
|  |  | Yes (440) | Varicella Zoster | 46 | Infection (unknown) | - GVHD post-DLI requiring intensive immune suppression - Aspiration pneumonia (could be enterococcus faecium found in blood) |
| DLI, donor lymphocyte infusion; GVHD, graft-versus-host disease | | | | | | |

**ST3. Events of Grade ≥3 viral infection/reactivation in patients who received TCD-haplo + ATIR101**

|  | **Events, n (%); Total=20*** | | | |
| --- | --- | --- | --- | --- |
| **Grade ≥3 viral infections** | **All Grade ≥3** | **Grade 3** | **Grade 4** | **Grade 5** |
| Epstein–Barr virus | 5 (25) | 4 (20) | 1 (5) | 0 (0) |
| Cytomegalovirus | 5 (25) | 5 (25) | 0 (0) | 0 (0) |
| Herpes (simplex/zoster) | 3 (15) | 3 (15) | 0 (0) | 0 (0) |
| Polyomaviruses (BK/JC) | 2 (10) | 1 (5) | 0 (0) | 1 (5) |
| Respiratory syncytial virus | 1 (5) | 1 (5) | 0 (0) | 0 (0) |
| Adenovirus | 2 (10) | 1 (5) | 0 (0) | 1 (5) |
| Rhinovirus A/B/C | 1 (5) | 1 (5) | 0 (0) | 0 (0) |
| Parvovirus | 1 (5) | 1 (5) | 0 (0) | 0 (0) |
| * 20 Grade ≥3 viral infections/reactivations occurring in 11 patients of the TCD-haplo + ATIR101 group over 2 years (viruses were not all typed).  TCD-haplo, T-cell-depleted haploidentical hematopoietic stem-cell transplantation. | | | | |

**ST4. Patients with infections at time intervals up to 2 years post HSCT (patients receiving TCD-haplo + ATIR101; N=23)**

|  | **From HSCT to  ATIR101 infusion**  **(N=23)** | **From ATIR101 infusion to 6 months after HSCT**  **(N=23)** | **From 6 months to 1 year after HSCT**  **(N=19)** | **From 1 year  to 2 years after HSCT**  **(N=14)** |
| --- | --- | --- | --- | --- |
| **Any infection, n (%)** |  |  |  |  |
| Patients | 17 (73.9) | 19 (82.6) | 13 (68.4) | 12 (85.7) |
| **Viral infection, n (%)** |  |  |  |  |
| Patients | 9 (39.1) | 16 (69.6) | 12 (63.2) | 12 (85.7) |
| Grade 1/2 | 9 (39.1) | 9 (39.1) | 9 (47.4) | 7 (50.0) |
| Grade ≥3 | 0 | 7 (30.4) | 3 (15.8) | 5 (35.7) |
| **Fungal infection, n (%)** |  |  |  |  |
| Patients | 8 (34.8) | 10 (43.5) | 5 (26.3) | 4 (28.6) |
| Grade 1/2 | 6 (26.1) | 8 (34.8) | 4 (21.1) | 2 (14.3) |
| Grade ≥3 | 2 (8.7) | 2 (8.7) | 1 (5.3) | 2 (14.3) |
| **Bacterial infection, n (%)** |  |  |  |  |
| Patients | 9 (39.1) | 8 (34.8) | 5 (26.3) | 7 (50.0) |
| Grade 1/2 | 3 (13.0) | 6 (26.1) | 3 (15.8) | 2 (14.3) |
| Grade ≥3 | 6 (26.1) | 2 (8.7) | 2 (10.5) | 5 (35.7) |
| For each patient, the worst severity of infection is shown. Severity was graded according to CTCAE version 4.0. Allocation of events to assessment periods is based on the onset date of the respective infection. N reflects the number of patients at the start of the assessment period. Infections with unknown causative agent are not included in this table.  HSCT, hematopoietic stem-cell transplantation; TCD-haplo, T-cell-depleted haploidentical HSCT. | | | | |

**References**

1. Simon R. Optimal two-stage designs for phase II clinical trials. *Controlled Clinical Trials* 1989; **10:** 1-10.

2. Przepiorka D, Weisdorf D, Martin P, Klingemann HG, Beatty P, Hows J *et al.* 1994 Consensus Conference on Acute GVHD Grading. *Bone Marrow Transplant* 1995; **15:** 825-828.

3. Filipovich AH, Weisdorf D, Pavletic S, Socie G, Wingard JR, Lee SJ *et al.* National Institutes of Health consensus development project on criteria for clinical trials in chronic graft-versus-host disease: I. Diagnosis and staging working group report. *Biol Blood Marrow Transplant* 2005; **11:** 945-956.

4. Holtan SG, DeFor TE, Lazaryan A, Bejanyan N, Arora M, Brunstein CG *et al.* Composite end point of graft-versus-host disease-free, relapse-free survival after allogeneic hematopoietic cell transplantation. *Blood* 2015; **125:** 1333-1338.
